# DNA Methylome Distinguishes Head and Neck Cancer from Potentially Malignant Oral Lesions and Healthy Oral Mucosa

**DOI:** 10.3390/ijms21186853

**Published:** 2020-09-18

**Authors:** Nina Milutin Gašperov, Ivan Sabol, Ksenija Božinović, Emil Dediol, Marinka Mravak-Stipetić, Danilo Licastro, Simeone Dal Monego, Magdalena Grce

**Affiliations:** 1 Division of Molecular Medicine, Ruđer Bošković Institute, 10000 Zagreb, Croatia; isabol@irb.hr (I.S.); ksenija.bozinovic@irb.hr (K.B.); 2 Department of Maxillofacial Surgery, School of Medicine, Clinical Hospital Dubrava, University of Zagreb, 10000 Zagreb, Croatia; edediol@mef.hr; 3 Department of Oral Medicine, School of Dental Medicine, University of Zagreb, 10000 Zagreb, Croatia; mravak@sfzg.hr; 4 ARGO Open Lab Platform for Genome sequencing, AREA Science Park, Padriciano, 99, 34149 Trieste, Italy; danilo.licastro@areasciencepark.it (D.L.); simeone.dalmonego@cbm.fvg.it (S.D.M.)

**Keywords:** DNA methylation, head and neck squamous cell carcinoma (HNSCC), potentially premalignant oral lesions, healthy oral mucosa, human papillomavirus (HPV)

## Abstract

There is a strong need to find new, good biomarkers of head and neck squamous cell carcinoma (HNSCC) because of the bad prognoses and high mortality rates. The aim of this study was to identify the potential biomarkers in HNSCC that have differences in their DNA methylome and potentially premalignant oral lesions, in comparison to healthy oral mucosa. In this study, 32 oral samples were tested: nine healthy oral mucosae, 13 HNSCC, and 10 oral lesions for DNA methylation by the Infinium MethylationEPIC BeadChip. Our findings showed that a panel of genes significantly hypermethylated in their promoters or specific sites in HNSCC samples in comparison to healthy oral samples, which are mainly oncogenes, receptor, and transcription factor genes, or genes included in cell cycle, transformation, apoptosis, and autophagy. A group of hypomethylated genes in HNSCC, in comparison to healthy oral mucosa, are mainly involved in the host immune response and transcriptional regulation. The results also showed significant differences in gene methylation between HNSCC and potentially premalignant oral lesions, as well as differently methylated genes that discriminate between oral lesions and healthy mucosa. The given methylation panels point to novel potential biomarkers for early diagnostics of HNSCC, as well as potentially premalignant oral lesions.

## 1. Introduction

Head and neck squamous cell carcinoma (HNSCC), which encompasses tumors of the oral and nasal cavities, paranasal sinuses, pharynx, and larynx, is the 6th most frequent malignancy in the world, with over 650,000 new cases diagnosed each year [1]. Although the overall survival of patients with oral cancer has improved during the past 20 years, it has only improved marginally; mainly due to the advanced clinical stage at diagnosis and the high rates of treatment failure associated with this advanced disease [2].

The most important risk factors identified so far for HNSCC are excessive tobacco [3,4] and alcohol consumption [5,6,7], together with high-risk types of human papillomavirus (HPV) [8,9,10]. Although the global incidence of HNSCC is declining, the incidence of HPV related HNSCC, especially oropharyngeal and oral squamous cell carcinoma (OPSCC and OSCC, respectively) is rapidly increasing over the last few decades [11]. Recent findings emphasize the importance of epigenetic changes, such as DNA methylation and alterations including micro RNAs (miRNA), in HNSCC progression and implicate the very role of tobacco and alcohol [12], as well as HPV [13] in those changes.

The HNSCCs are one of the cancer types with the worst prognosis and with a high mortality of patients, hence, there is a strong need to find new biomarkers of this disease [14,15]. The most appropriate biomarkers would be those pointing out changes on the cellular level before carcinoma can be detected or even before carcinoma occurrence. The epigenetic biomarkers, such as methylated genes could efficiently point to changes before cancers can be clinically detected and help us better understand tumorigenesis and hopefully improve cancer treatment and prevention [16,17]. Some of these potential biomarkers could also differentiate between the groups of potentially premalignant oral lesions that show possible premalignant transformation, such as oral lichen planus (OLP) and oral lichenoid lesions (OLL), whose treatment is different from each other despite their high clinical and histopathological similarities [18,19,20]. Namely, OLP is a chronic immunological mucocutaneous disorder of unknown etiology, while OLL is usually of known etiology, being a lichenoid contact stomatitis [21].

DNA methylation is the most studied epigenetic change in human diseases, especially cancer because it is apparently stable under most storage conditions, even as histological preparations [22]. Altered DNA methylation is one of the possible factors associated even with the HNSCC development. The focus of this study was to explore DNA methylation changes that are significantly deregulated in HNSCC samples, particularly OPSCC and OSCC, and potentially premalignant oral lesions (such as OLP and OLL) in comparison to healthy oral mucosa. Identifying DNA methylome differences by means of the Infinium MethylationEPIC BeadChip array (Illumina, San Diego, California, United States) on the level of methylated genes, gene promoters, and individual 5′-cytosine-phosphate-guanine-3′ (CpG) sites between HNSCC, potentially premalignant oral lesions, and healthy oral mucosa enables us to suggest novel potential biomarkers for the identification of HNSCC and potentially premalignant oral lesions.

## 2. Results

### 2.1. HPV Status

HPV DNA was found in nine of 32 samples, of which are five cancer samples, three potentially premalignant oral lesions samples, and one control sample (healthy mucosa) (Figure S1). HPV 16 was found in all five HPV positive cancer samples. HPV 58 was found in one OLP sample positive, while undetermined HPV types were found in one OLP, one OLL, and one control sample.

### 2.2. Overall DNA Methylation Findings

After pre-processing, normalization, and batch correction of Infinium MethylationEPIC BeadChip data in ChAMP, 679,851 probes were retained for analysis by the RnBeads package. The inclusion criteria was the value of the mean difference across all sites in a region (mean.mean.diff; MMD), the highest and the lowest values from the Illumina assay data. The cut-off values were: FDR adjusted *p*-value ≤ 0.05 and mean methylation difference ≥ |0.2|. For every set of data, the list of first 15 genes, the best significantly differentiated between groups of samples in methylation, i.e., hyper- or hypomethylated, is presented (the mean methylation difference was ≥ |0.44|). The whole list of genes, besides a large number of defined genes includes not annotated (NA) genes, pseudogenes, and RNA genes (could be provided upon request).

**Figure 1:**
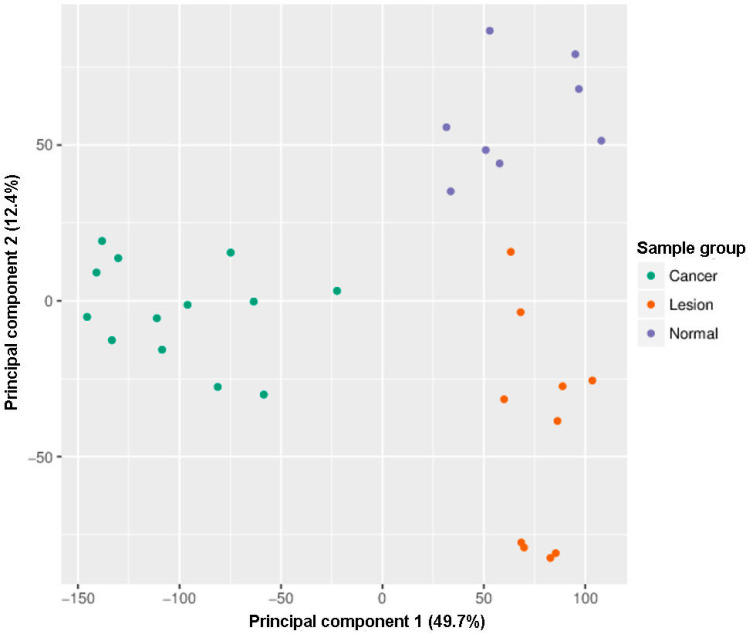
Infinium MethylationEPIC BeadChip findings: Samples grouping by the principal component. Green: Head and neck squamous cell carcinoma (HNSCC) samples, oropharyngeal cancer (*n* = 6) and oral cancer (*n* = 7), orange: Oral lesions, OLP (*n* = 8) and OLL (*n* = 2), violet: Healthy oral mucosa (*n* = 8).

**Figure 2:**
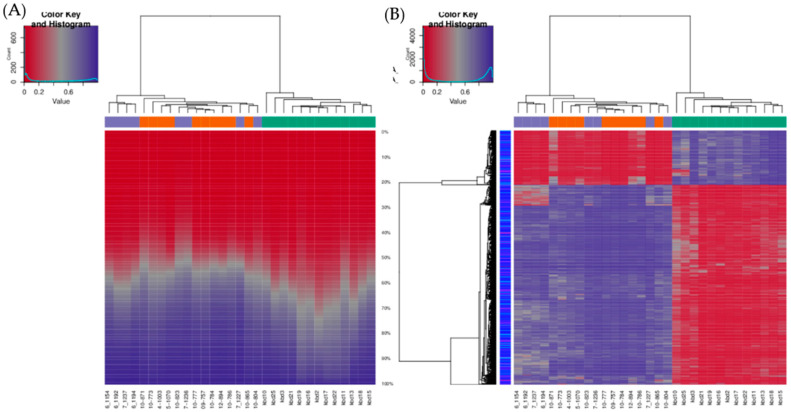
Infinium MethylationEPIC BeadChip findings: Hierarchical clustering of samples based on all methylation values. (**A**) The heatmap displays methylation percentiles per sample. (**B**) The heatmap displays only selected sites/regions with the highest variance across all samples. Green: HNSCC samples, oropharyngeal cancer (*n* = 6) and oral cancer (*n* = 7), orange: Oral lesions, OLP (*n* = 8) and OLL (*n* = 2), violet: Healthy oral mucosa (*n* = 8).

**Figure 3:**
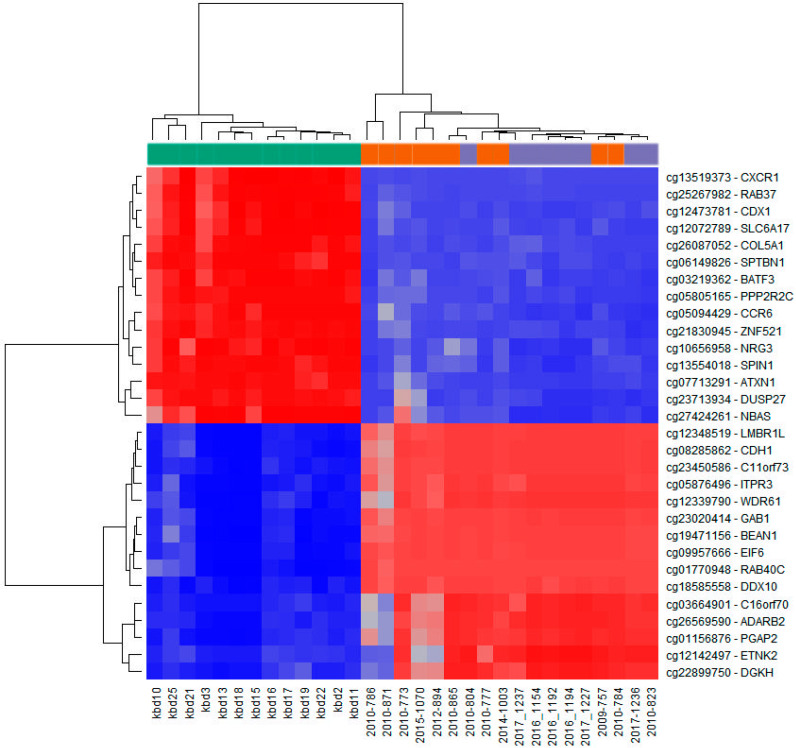
Infinium MethylationEPIC BeadChip findings: Heatmap representation of samples clustering for the top fifteen hypermethylated (blue) and top fifteen hypomethylated (red) CpG sites in cancer tissue compared to control healthy tissue (according to the methylation difference value; Table 3 and Table 4). Left side (green): HNSCC samples (*n* = 13; kbd10–kbd11); right side (violet): Healthy oral mucosa (*n* = 8; 2010-804, 2017-1237–2017-1227, 2017-1236, 2010-823); middle (orange): Oral lesions (*n* = 10; 2010-786–2010-865, 2010-777, 2014-1003, 2009-757, 2010-784).

Within the exploratory analysis, the principal component analysis (PCA) showed that all samples clustered within three distinct clusters: Cancer, lesions, and controls (Figure 1). One healthy oral mucosa sample of 32 oral samples analyzed by the Infinium MethylationEPIC BeadChip was automatically excluded during filtering and normalization steps (Figure 1, Figure 2 and Figure 3, and Figure S1).

The unsupervised hierarchical clustering analysis based on all investigated methylation sites across the genome and all methylation values showed good clustering of cancer samples on one side, however control samples and potentially premalignant oral lesions were clustered together on the other side (Figure 2A). The situation was similar when visualizing only the top 1000 most variable positions (Figure 2B). As expected, global cancer hypomethylation can be seen in both figures as more CpG sites exhibit lower methylation values in cancer cluster. The heatmap representation of samples clustering for the top fifteen hypermethylated and the top fifteen hypomethylated CpG gene sites in cancer tissue compared to control healthy tissue also showed good clustering of cancer samples on one side and control samples on the other, while most of the potentially premalignant oral lesions were clustered between those two groups (Figure 3).

### 2.3. Differentially Methylated Gene Promoters in HNSCC Tissue Compared to Control Tissue

The top fifteen genes significantly hypermethylated in their promoters in cancer tissues in comparison to healthy tissues are: *GPRC5D*, *TMPRSS11B*, *PIAS2*, *ARG1*, *SRPK2*, *AADACL2*, *RGPD4*, *SPRR3*, *DEGS1*, *TXNDC8*, *SH3TC1*, *ZPLD1*, *FBXO2*, *ATG16L1,* and *GRHL1* (Table 1). The FDR adjusted *p*-value was < 0.05 and the difference in the average methylation value between 0.78 and 0.44. The hypermethylated genes are mostly involved in different cellular enzymatic reactions and autophagy.

The top fifteen genes that were significantly hypomethylated in their promoters in cancer tissues in comparison to healthy tissues are: *TRBC2*, *DGAT2*, *ALG1L*, *PDE4D*, *TRDC*, *DNAJC6*, *IGKV3-20*, *TMEM150B*, *LAIR2*, *UBQLN3*, *ANKFN1*, *MS4A1*, *CCT8L2*, *SPOCK1,* and *IGHV4-39* (Table 2). The FDR adjusted *p*-value was < 0.05 and the difference in the average methylation value between −0.80 and −0.61. The hypomethylated genes are mainly involved in the immune response.

### 2.4. Differentially Methylated CPG Sites in HNSCC Tissue Compared to Control Tissue

From the complete list of differentially methylated CpG sites, only those falling within or near defined genes were selected, while all other sites for which the biologic relevance could not be evaluated were excluded (Figure 3). Thus, the top fifteen significantly hypermethylated sites in cancer tissues in comparison to healthy tissues are: *LMBR1L*, *CDH1*, *EIF6*, *C16orf70*, *ETNK2*, *C11orf73*, *ADARB2*, *GAB1*, *ITPR3*, *WDR61*, *PGAP2*, *DDX10*, *DGKH*, *RAB40C,* and *BEAN1* genes (Table 3). The FDR adjusted *p*-value was < 0.05 and the difference between mean methylation values across sites between 0.93 and 0.89. The hypermethylated genes are mostly involved in translation processes and cellular growth, transformation, and proliferation.

The top fifteen genes significantly hypomethylated on different sites across the genome (5′UTR, 3′UTR, TSS1500, TSS200, 1st exon, exon body) in cancer tissues in comparison to healthy tissues are: *ATXN1*, *PPP2R2C*, *CCR6*, *RAB37*, *DUSP27*, *ZNF521*, *SLC6A17*, *SPIN1*, *CXCR1*, *SPTBN1*, *NBAS*, *NRG3*, *COL5A1*, *CDX1,* and *BATF3* (Table 4). The FDR adjusted *p*-value was < 0.05 and the difference between mean methylation values across sites between −0.96 and −0.89. The hypomethylated genes are mostly involved in transcriptional and immune regulation.

### 2.5. Aberrant Methylation in Potentially Premalignant Oral Lesions

The aberrant methylation in gene promoters and CpG sites within defined genes in HNSCC tissue compared to potentially premalignant oral lesions, OLP and OLL, and in oral lesions compared to healthy tissue are shown in Table 1, Table 2, Table 3, and Table 4, respectively.

The top fifteen genes significantly hypermethylated in their promoters that could distinguish HNSCC from potentially premalignant oral lesions are: *RAD51B*, *BARX2*, *SLC5A10/FAM83G*, *NINL*, *NSMCE2*, *PGAP2*, *INO80C*, *IL34*, *ZNF516*, *GFOD2*, *PARD3*, *MCEE*, *POLM*, *ASPG,* and *TBC1D2* (Table 1). The top fifteen genes significantly hypomethylated in their promoters in HNSCC compared to potentially premalignant oral lesions are: *ART4*, *EPB41L3*, *ESRRG*, *ENPP1*, *GNG7*, *PAPSS2*, *NGEF*, *HIPK4*, *GPR158*, *GSG1L*, *SMPD3*, *GDF2*, *RERE*, *CDH13,* and *HS3ST4* (Table 2).

The top fifteen genes significantly hypermethylated (*SLC5A10*, *TBC1D2*, *SH3BP5L*, *VANGL1*, *DLEC1*, *TGOLN2*, *CTBP2*, *PPP1CB*, *VPS52*, *MEPCE*, *HDAC4*, *ARAP1*, *TCF20*, *NDUFS7*, and *GATAD2A*) and the top fifteen genes significantly hypomethylated in their promoters (*ART4*, *ENPP1*, *GNG7*, *PKD1L3*, *PLXNC1*, *CAMK2B*, *CACNA1S*, *SCGB1D1*, *VPS13D*, *DLGAP4*, *LRP1B*, *COL2A1*, *SLC24A3*, *TBC1D8,* and *ABCC8*) that could distinguish potentially premalignant oral lesions from healthy oral mucosa are shown in Table 1 and Table 2. The top fifteen genes significantly hypermethylated (*GRIP1*, *MTMR10*, *RBM47*, *MPHOSPH9*, *FOXK1*, *SNX3*, *CIT*, *ZBTB38*, DRD3, *SPPL3*, *ZNF407*, *ADAMTSL1*, *GNAT3*, *L3MBTL3,* and *EEPD1*) and the top fifteen genes significantly hypomethylated (*PHACTR1*, *MARCH8*, *PPP1R1B*, *HDAC4*, *IL22RA2*, *CAMKK2*, *INPP5D*, *CSGALNACT1*, *GTDC1*, *IGSF3*, *HELZ*, *DEFA4*, *AK5*, *LHFPL2,* and *STK10*) on different sites across the genome in potentially premalignant oral lesions in comparison to healthy oral mucosa are shown in Table 3 and Table 4.

### 2.6. Validation Panel

Pyrosequencing was performed on a subset of samples tested by the Infinium MethylationEPIC BeadChip array (Illumina) for four gene promoters, namely *SPRR3*, *FBXO2* (hypermethylated in HNSCC tissue vs. control healthy tissue; Table 1), *TRDC* and *LAIR2* (hypomethylated in HNSCC tissue vs. control healthy tissue; Table 2) tested on four cancer samples and four control samples, each. The selection criteria were the role of these genes in biological processes as well as the findings in previous studies. *SPRR3* (Small Proline Rich Protein 3) and *FBXO2* (F-Box Protein 2) being largely investigated; *SPRR3* is involved in cornification, epidermis development, squamous cell differentiation, and peptide cross linking [23], while *FBXO2* is involved in the negative regulation of cell proliferation, cellular protein modification, and protein ubiquitination [24]. In addition, the hypomethylated genes in cancer are mostly involved in the immune response; *TRDC* (T Cell Receptor Delta Constant) being involved in recognizing foreign antigens, which have been processed as small peptides and bound to major histocompatibility complex (MHC) molecules at the surface of antigen presenting cells [25]. *LAIR-2* (Leukocyte Associated Immunoglobulin Like Receptor 2) related pathways belong to the innate immune system, and class I MHC mediated antigen processing, and presentation and immunoregulatory interactions [25].

For pyrosequencing validation, six amplifying PCR reactions (*SPRR3-1*, *SPRR3-2*, *TRDC-1*, *TRDC-2*, *LAIR2-1*, *LAIR2-2*, *FBXO2-1*) with six sequencing primers have been performed to cover four CpG sites for *SPRR3*, four CpGs for *FBXO2*, two CpGs for *TRDC,* and five CpGs for *LAIR2* gene. The overall pyrosequencing data for tested CpGs were in agreement with the methylation array data (Figure S2). However, statistical significance was only reached between HNSCC and the control samples in CpG1 and CpG3 of *SPRR3* gene (*p* = 0.01 in both cases) and CpG1 of *FBXO2* gene (*p* = 0.01).

### 2.7. Gene Set Enrichment Data

**Figure 4:**
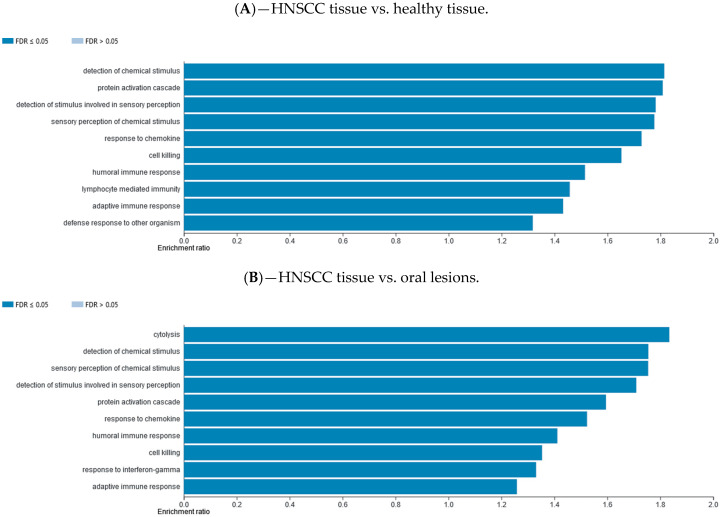

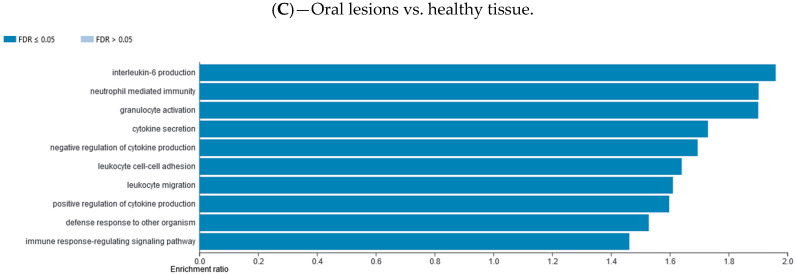
Over-representation enrichment analysis (ORA) of gene ontology (GO), biological processes, for consistently hypomethylated gene promoters and/or CpG sites in (**A**) HNSCC tissue compared to control healthy tissue, (**B**) HNSCC tissue compared to potentially premalignant oral lesions, and (**C**) potentially premalignant oral lesions compared to control healthy tissue. The top 10 categories are shown; the fold discovery rate (FDR) adjusted significance (colored bar) is in each shown case ≤0.05.

Differentially methylated gene promotor regions were analyzed using the WebGestalt functional enrichment analysis web tool to determine whether the affected genes are enriched for specific sets of functions or pathways. Methylation data were explored with two different analysis approaches available, over-representation enrichment analysis (ORA) and gene set enrichment analysis (GSEA). The ORA of gene ontology (GO) data (biological processes) for consistently hypomethylated gene promoters and/or CpG sites in one group of samples vs. the other group are presented in Figure 4. The ORA of GO data (biological processes) for consistently hypermethylated gene promoters and/or CpG sites in one group of samples vs. the other group are presented in Supplementary Materials file (Figure S8). The top 10 GO categories are presented. The ORA analysis of KEGG pathway for hypomethylated and hypermethylated gene promoters and/or CpG sites in one group of samples vs. the other group are presented in Supplementary Materials file (Figures S7 and S9, respectively). The top 10 KEGG categories are presented. The GSEA analysis of GO biological processes for hypomethylated and hypermethylated gene promoters and/or CpG sites in one group of samples vs. the other group are presented in Supplementary Materials file (Figures S10 and S12, respectively). The GSEA analysis of KEGG pathway for hypomethylated and hypermethylated gene promoters and/or CpG sites in one group of samples vs. the other group are presented in Supplementary Materials file (Figures S11 and S13, respectively).

### 2.8. External Database Validation

Our gene promoters’ methylation findings were compared to TCGA Illumina HISeq RNAseq data of TCGA-HNSC project. The RNAseq estimation of expression for the top fifteen hypermethylated and top fifteen hypomethylated gene promoters in our results were visualized in Wanderer, an interactive viewer. The TCGA dataset included 497 tumor and 43 normal tissue samples. There was a good agreement between our gene promoters’ methylation data and the gene expression data (Table S1, Figures S3–S6). Out of the total of top fifteen hypermethylated gene promoters in our study, ten were found to be either under-expressed or hypermethylated in TCGA cancer cases, as expected, while one had no measurable expression and only a single CpG site in Illumina 450K DNA methylation array. From the top fifteen hypomethylated gene promoters in our study, twelve were also found to be either over-expressed or hypomethylated in TCGA data, with the remaining three lacking annotated data or probes in Illumina 450K DNA methylation array.

## 3. Discussion

The aim of this study was to investigate DNA methylome in HNSCC and potentially premalignant oral lesions, as well as in healthy oral tissue, and to identify the best genes that are differentially methylated in gene promoters or specific sites among those groups of samples. We found that components of different cellular pathways are differently methylated in HNSCC in comparison to healthy oral tissue as well as potentially premalignant oral lesions.

Surprisingly, we could not observe any grouping of samples in accordance with their HPV status, thus subsequent analysis focused only on the sample origin. The lack of HPV specific differences could possibly be explained by a limited number of HPV positive samples (nine of 32). Furthermore, HPV is known to be more associated with oropharyngeal tumors than oral cavity cancerogenesis [26]. Another possible explanation is the particularity of the Croatian population where smoking and drinking are almost equally present in HPV positive and HPV negative OPSCC patients shown in our previous study [27]. On the other hand, the study of Lechner et al. with Infinium HumanMethylation450 BeadChips (Illumina) showed unsupervised clustering over the methylation variable positions of samples in accordance with the HPV status. Nevertheless, they showed that HPV positive tumors are heterogeneous, which led to the identification of a candidate CpG island methylator phenotype in a sub-group of HPV positive tumors [28].

Herein, the top fifteen genes with a significant promoter hypermethylation in cancer tissues in comparison to control healthy tissues, named *GPRC5D*, *TMPRSS11B*, *PIAS2*, *ARG1*, *SRPK2*, *AADACL2*, *RGPD4*, *SPRR3*, *DEGS1*, *TXNDC8*, *SH3TC1*, *ZPLD1*, *FBXO2*, *ATG16L1*, and *GRHL1* are mostly involved in different cellular enzymatic reactions and in autophagy (Table 1). For example, the expression of *SPRR3* (Small Proline Rich Protein 3) was found to be associated with tumor cell proliferation and invasion in glioblastoma multiforme. Liu et al. (2013) found, contrary to our findings, that *SPRR3* hypomethylation was associated with the clinical outcome in glioblastoma multiforme patients [29]. In an anatomically more similar context, *SPRR3* was frequently downregulated in OPSCC where it probably suppresses tumorigenicity [30]. In our study, we selected the promoter of the *SPRR3* and *FBXO2* genes for validation by pyrosequencing and found that both methods agree on the direction of methylation deregulation, which is hypermethylation of the gene promotor.

The top fifteen genes in cancer tissues that were found in this study to be significantly hypomethylated in their promoters in comparison to control healthy tissues (*TRBC2*, *DGAT2*, *ALG1L*, *PDE4D*, *TRDC*, *DNAJC6*, *IGKV3-20*, *TMEM150B*, *LAIR2*, *UBQLN3*, *ANKFN1*, *MS4A1*, *CCT8L2*, *SPOCK1,* and *IGHV4-39*) are mainly involved in the immune response, i.e., *IGHV4-39* (antigen recognition gene), *IGKV3-20* (immunoglobulin receptor binding gene), *LAIR2* (innate immune response gene), *MS4A1* (differentiation of B cells gene), *TRBC2* and *TRDC* (both T cell receptor genes). Indeed, the HNSCC are known for their immune-suppressive character allowing tumor evasion and escape from the immune surveillance, which probably can be associated with the methylation of immune-response related genes [31]. Here again, from the list of genes with hypomethylated promoters we selected *LAIR2* and *TRDC* for validation and, as expected, both gave comparable results on pyrosequencing.

The top fifteen significantly hypermethylated genes, named *LMBR1L*, *CDH1*, *EIF6*, *C16orf70*, *ETNK2*, *C11orf73*, *ADARB2*, *GAB1*, *ITPR3*, *WDR61*, *PGAP2*, *DDX10*, *DGKH*, *RAB40C*, and *BEAN1* on different gene sites (mostly in 5′UTR and body) in cancer tissues in comparison to control healthy oral tissues are mostly involved in translational processes and cellular growth, along with transformation and proliferation. Among them, *CDH1*, *ETNK2*, *ADARB2,* and *RAB40C* are found to be aberrantly methylated in different cancers [32,33,34,35]. For instance, altered methylation levels of *CDH1* (Cadherin 1), whose loss contributes to cancer progression by increasing proliferation, invasion, and/or metastasis are recorded in oral cavity [32], oral [36], and in cervical cancer [37]. The study of Strzelczyk et al. [32] reported a significantly higher methylation level of *CDH1* in tumor tissues compared to surgical margins (57% vs. 25% *p* < 0.001) in patients with oral cavity cancer. The meta-analysis of the gene promoter hypermethylation in oral cancer, that included 29 studies of which 13 were about *CDH1* methylation, showed a significant correlation of *CDH1* hypermethylation with oral cancer risk [36]. Moreover, in the meta-analysis of Liu et al. [37] on patients with cervical carcinoma, *CDH1* promoter methylation was significantly higher in cancer than in cervical intraepithelial neoplasia lesions and healthy cervical tissues.

The first fifteen genes that were significantly hypomethylated on different sites across the genome in cancer tissues in comparison to control healthy tissues, named *ATXN1*, *PPP2R2C*, *CCR6*, *RAB37*, *DUSP27*, *ZNF521*, *SLC6A17*, *SPIN1*, *CXCR1*, *SPTBN1*, *NBAS*, *NRG3*, *COL5A1*, *CDX1*, and *BATF3* are mostly involved in transcriptional and immune regulation. Among this group of genes, aberrantly methylated in other human cancers were *CCR6* in oral cancer [38] and chronic lymphocytic leukemia [39], *RAB37* in lung cancer [40], *ZNF521* in breast cancer [41], and *CDX1* in gastric cancer [42], esophageal SCC [43], and in colon cancer [44]. The genes involved in the immune regulation could belong to the tumor-infiltrating immune cells or tumor-infiltrating lymphocytes, which are often associated with better clinical outcomes. Thus, the aberrantly methylated gene *CCR6* (C-C Motif Chemokine Receptor 6), which regulates the migration and recruitment of dendritic and T cells during inflammatory and immunological responses, was also found in human OSCC [38]. Lee et al. [38] concluded that hypomethylation of this gene may play an important role in the recruitment or retention of CCR6+ Treg cells into the OSCC inflammatory microenvironment at the early stage of tumor progression. In addition, a genome-wide DNA methylation analysis of chronic lymphocytic leukemia patients in comparison to healthy donors identified the differently methylated *CCR6* gene, among other immune regulatory genes [39]. In addition, in their study, Kim et al. presented that the majority of hypomethylated gene sets identified across multiple cancer (breast, lung cancer, colorectal, myeloma, glioblastoma, ovarian, kidney and stomach cancer) studies were immune-related, suggesting DNA methylation-driven cancer cell invasion and tumorigenesis across various types of cancer [45].

The external validation of our top thirty differentially methylated gene promoters in HNSCC vs. control tissue with gene expression data in human cancer through Wanderer, an interactive viewer, gave a very good agreement. In summary, the majority of hypermethylated gene promoters in HNSCC in our study (10 of 15) were found to be either under-expressed or hypermethylated in TCGA cancer cases. In addition, from the top 15 hypomethlylated gene promoters in our study, 12 were also found to be either over-expressed or hypomethylated in TCGA data.

Of particular interest in the HNSCC diagnostic, clinical prognosis and/or risk assessment could be the methylation of *CDH1*, which was also previously described as a possible biomarker for the early detection and treatment of HNSCC [32,36,46,47]. We also validated the *CDH1* gene promoter in the same groups of cancer samples and healthy controls by Methylation-Specific PCR (MSP), and the findings are in concordance with the Infinium MethylationEPIC BeadChip array findings (data not presented). The majority of HNSCC samples (85%) were methylated in the *CDH1* gene promoter by MSP, while only 21% of healthy control samples were methylated in the same gene promoter. In this study, we found the *CDH1* gene to be significantly hypermethylated on specific sites in the genome (body) on a high second place in cancer tissues in comparison to control tissues. The same gene (*CDH1*) is also among the top fifteen genes that are significantly hypermethylated on different sites across the genome in cancer tissues compared to lesions. The *CDH1* gene encodes E-cadherin, a classical cadherin of the cadherin superfamily that is involved in mechanisms regulating cell-cell adhesions, mobility, and proliferation of epithelial cells. It is recognized as a tumor suppressor gene; the loss of function of this gene is thought to contribute to cancer progression by increasing proliferation, invasion, and/or metastasis [25]. Hence, we showed herein that hypermethylation on specific CpGs within the *CDH1* gene could be a good biomarker of HNC and a possible option to distinguish HNSCC from potentially premalignant oral lesions and from healthy oral mucosa as well.

Two other genes that are present in the top fifteen most significantly hypermethylated in gene promoter regions in cancer tissues compared to lesions, and in lesions compared to control healthy tissues are the *SLC5A10* (Solute Carrier Family 5 Member 10) and the *TBC1D2* (TBC1 Domain Family Member 2) gene. The *SLC5A10* gene is a member of the sodium/glucose transporter family, while the *TBC1D2* gene acts as GTPase-activating protein for RAB7A, involved in cadherin degradation and cell-cell adhesion. Notably, two out of the three genes, whose hypermethylation may be of particular importance in HNSCC diagnostic, *CDH1* and *TBC1D2*, are involved in cadherin regulation of cell-cell adhesion. Suppression of cadherins in HNSCC leads to cells escaping from the contact-dependent growth, which develop a migratory phenotype with low differentiation stage, suggesting that cadherins contribute to the transformation steps [48]. The two genes from the group, *SLC5A10* and *TBC1D2*, could also be considered as possible good methylation biomarkers to distinguish oral potentially premalignant lesions from healthy oral tissue.

Unexpectedly, the overlap of significant findings on the CpG site and gene promoter levels in the whole study was non-existent, probably because most of the top-rated promoters included only one or rarely few sites in the analysis. Further, there is no evidence in the literature on this issue to conform or refute these observations.

Using the WebGestalt functional enrichment analysis web tool we assessed gene enrichment for specific sets of functions or pathways and networking. Indeed, the over-representation enrichment analysis (ORA) of GO non-redundant biological processes for differentially methylated gene promoters presented an implication of mostly immune response and cellular defense response pathways, as well as cell-cell adhesion.

The current study is the first to implement the Infinium MethylationEPIC BeadChip assay on a well-defined set of clinical samples encompassing the whole possible spectrum from healthy tissue to cancer. To our knowledge, this is the first such study focused on HNSCC, oral lesions, and healthy tissue together. In addition, the power of the study relies on prospectively collected fresh samples with minimum delays between sample collection and processing. However, for that reason the limitation of the study might be the possibility that infiltrating immune cells could be present in tumor tissues. Indeed, we performed the analysis of tumor purity by leukocytes unmethylation for the purity (LUMP) method [49] and assessed the variability of the tissue cellular composition (Figure S14): Different amounts of infiltrating immune cells for every group of samples, with a significantly lower proportion (*p* < 0.05) of the same in a healthy oral tissue (20%) than in HNSCC (56%) and lesions’ samples (65%). In addition, one of the strengths of this study was the simultaneous microarray testing in the same analysis of different tissues, cancer, oral lesions, and healthy tissue. On the other hand, the study was limited by anatomical differences in the sample material, namely both healthy and potentially premalignant oral lesions samples were mostly derived from the oral cavity, where potentially premalignant oral lesions usually originate, while cancer samples included both oral, and oropharyngeal cancer. Another possible limitation was the age of participants as cancer usually develops later in life, while the average age of controls and patients with potentially premalignant oral lesions was lower (43 vs. 53 years). We attempted to adjust for this by including age as a covariate. For the future study, we plan to collect also the healthy oral mucosa from the same patient to investigate possible differences. Overall, our study has demonstrated a significant overlap with current knowledge, which together with successful validation of the data by pyrosequencing confirms the reliability of the underlying data and strengthens its results.

## 4. Materials and Methods

### 4.1. Study Group

Healthy oral mucosa samples were collected from healthy subjects in the School of Dental Medicine, University of Zagreb, Croatia, during a regular process of teeth extraction from 2010 to 2017. Oral samples of potentially premalignant oral lesions, OLP and OLL, were taken cytologically in the School of Dental Medicine, University of Zagreb, Croatia, from 2008 to 2016. HNSCC samples were collected in the Clinical Hospital Dubrava, Zagreb, Croatia, from 2014 to 2018. The study group comprised 32 oral samples: nine healthy oral mucosa, 10 potentially premalignant oral lesions (eight OLP and two OLL), and 13 HNSCC (six oropharyngeal cancers and seven oral cancers). The median age among patients with HNSCC was 57 years, while amongst patients with potentially premalignant oral lesions and control healthy mucosa was slightly lower, 43 and 53 years, respectively. There were 10 men and three women with HNSCC, four men and six women with potentially premalignant oral lesions, and four men and five women with healthy oral mucosa and without drinking and smoking history. Fresh samples were collected with the cytobrush, stored in appropriate buffers for further analysis, HPV testing, and DNA methylation analysis.

### 4.2. DNA Preparation

The extracted DNA from oral specimens was processed without initial knowledge of patients’ data. DNA was isolated using the BioRobot EZ1 (Qiagen, Hilden, Germany) system according to the manufacturer’s instructions. After DNA extraction, the purified DNA was dissolved in 50–100 μL of tri-distillate sterile water and stored at −20 °C until further analysis. The quality and integrity of the samples were evaluated on a NanoPhotometer (Implen GmbH, München, Germany), and samples with the ratio A260/280 between 1.7–1.9 were included in the study [50].

### 4.3. HPV Detection and Typing

HPV testing is previously described [51,52]. Briefly, three sets of consensus primers for HPV detection were used: PGMY09/PGMY11, L1C1/L1C2-1/L1C2-2, and GP5+/GP6+. The quality of the isolated DNA was confirmed by amplification of the β-globin gene using PC04/GH20 primers in a multiplex polymerase chain reaction (PCR) with PGMY primers. Type-specific (TS) primers for HPV types 6/11, 16, 18, 31, 33, 45, 52, and 58 were used for HPV typing according to Milutin-Gasperov et al. [51]. Aliquots of each PCR product (10 μL) were analyzed by a 2% agarose gel electrophoresis and stained with Midori Green Advance dye. The amplified products were visualized by UV irradiation of the gels using the UVItec Cambridge (Alliance 4.7) imaging system. HPV positive samples that were not positive for TS-PCR but positive for consensus primer amplification were defined as undetermined HPV type.

### 4.4. Methylation Array Analysis

The Infinium MethylationEPIC BeadChip array (Illumina), which integrates a total of 863,904 CpG loci, together with 2932 non-CpG loci and 59 single nucleotide polymorphisms (SNPs), superseded the HM450 array, while still containing more than 90% of the original HM450 probes. Additional probes included in the new version of the array greatly increased the power of this microarray to study enhancer/regulatory regions [53]. Briefly, approximately 1–2 µg of DNA from cancer and oral samples were modified with sodium bisulfite using the EZ DNA Methylation Kit (Zymo Research, Irvine, CA, USA) and then purified according to the manufacturer’s instructions. After bisulfite treatment, 180–200 ng DNA was subjected to the whole genome amplification (WGA) and enzymatic digestion with the Infinium MethylationEPIC BeadChip kit reagents. The hybridization of the samples on the BeadChips and washing procedures followed the standard manufacturer’s protocol. The iScan System (Illumina) was used to read the BeadChips.

### 4.5. Data Processing and Statistical Analysis

Raw data obtained by iScan readout was imported to and analyzed within R using ChAMP (version 2.9.10) [54] and RnBeads packages (version 1.10.7; integrated software package for the analysis and interpretation of DNA methylation data) [55]. Briefly, data were imported to the Chip Analysis Methylation Pipeline (ChAMP) pre-processed, and normalized with the Peak Based Correction (PBC) method [56]. Subsequently, the Combat method [57] within ChAMP was used to adjust for batch effects. The resulting normalized and batch-corrected rnb.set was imported to the RnBeads package for subsequent exploratory and differential methylation analysis and customized visualization. RnBeads uses the limma normalization method (linear models for microarray data) [58] for differential methylation assessment between pairs of groups and herein the calculations were performed while adjusting for patient age and gender as covariates. In addition to analyzing differential methylation between groups on the individual CpG site level, all analyses were performed on the predefined gene and promoter levels by selecting appropriate region.types options within the RnBeads package.

### 4.6. Differentially Methylated Gene Promoters and Individual CPG Sites

The resulting differentially methylated promoter or CpG site lists were further filtered by selecting only promoters or sites with false discovery rate (FDR) adjusted *p*-values ≤ 0.05 and mean methylation difference ≥ |0.2|. For the top 15 candidates presented in Table 1, Table 2, Table 3 and Table 4, a more stringent differential methylation value of ≥ |0.44| is presented (between cancer and control healthy tissues, cancer and potentially premalignant oral lesions, and potentially premalignant oral lesions and control healthy tissues). The filtered tables contain the information on chromosome locations, and relation to any nearby CpG islands. Promoter level data additionally contains information about the number of included CpG sites and average GC content for the region.

### 4.7. Validation of Methylation by Pyrosequencing

Pyrosequencing assays were developed for the following genes: *SPRR3*, *FBXO2*, *TRDC*, and *LAIR2*. The PCR and sequencing primers were designed using the PyroMark Assay Design software, version 2.0.1.15 (Qiagen) to assess individual CpG sites of depicted genes from the Infinium MethylationEPIC BeadChip array analysis. All primers were purchased from Macrogen (Macrogen, Seoul, South Korea). The primer sequences, amplicon sizes, and the optimal annealing temperatures are indicated in Table S1. The analysis was performed on four control healthy and four HNSCC tissues (two HPV positive and two HPV negative), which were already tested by the Infinium MethylationEPIC BeadChip array. Briefly, approximately 500 ng of extracted DNA was used for the bisulfite treatment performed with the EZ DNA Methylation Kit, according to the instructions by the manufacturer, and eluted in a 20 µL elution buffer (Zymo Research). The PCR reactions were performed according to the PyroMark PCR protocol (Qiagen) in a total volume of 30 µL. Briefly, 0.10 µmol/L of each primer, 1.5 mM MgCl_2_, PyroMark PCR Master mix (Qiagen), Coral Load (Qiagen), and 50 ng of bisulfite treated template DNA were added to the PCR reaction and performed in a thermocycler (Veriti, 96 Well Thermal Cycler, Applied Biosystems, Foster City, California, USA). The program was as follows: Initial denaturation of 1 min at 95 °C, followed by 45 cycles of 30 s denaturation at 95 °C, specific annealing temperature for each primer pair (Table S1) for 30 s, and extension for 30 s at 72 °C with the final extension for 10 min at 72 °C. Pyrosequencing was performed using a PyroMark Q24 Reagent Kit and a PyroMark Q24 system (Qiagen) as described previously by Mikeska et al. [59]. The nucleotide addition order was optimized by the PyroMark Q24 Software (Qiagen) and the results were automatically analyzed using the same software. The percentage of methylation for each CpG island between the two sample groups (cancer vs. controls) was compared and *p*-values were determined using the *t*-test.

### 4.8. Gene Set Enrichment Analysis

The list of differentially methylated gene promotor regions was assessed to determine whether the affected genes are enriched for specific sets of functions or pathways. However, for the analysis, only those regions with assigned RefGene names indicating nearby or overlapping genes were selected. To make the analysis more stringent, only promotors with at least two CpG sites were included. The analysis was done using the WebGestalt functional enrichment analysis web tool [60]. Methylation data were explored with two different analysis approaches available, over-representation enrichment analysis (ORA) and gene set enrichment analysis (GSEA). For the ORA and GSEA analysis, the gene ontology—biological process (no-redundant) and KEGG (Kyoto Encyclopedia of Genes and Genomes) pathway databases were chosen. For ORA, the reference gene set was set to the whole genome, since many differentially methylated regions were related to miRNA and other non-coding sequences. For GSEA, gene promotors were ranked according to the Log2 of the mean difference and this data were supplied in addition to the gene symbol.

### 4.9. External Validation of Differentially Methylated Gene Promoters

Our gene promoters’ methylation findings were compared to Illumina HISeq RNAseq data of the TCGA-HNSC project through Wanderer (http://maplab.imppc.org/wanderer/), an interactive viewer to explore DNA methylation and gene expression data in human cancer [61]. The RNAseq estimation of expression for the top fifteen hypermethylated and top fifteen hypomethylated gene promoters in our results were visualized in Wanderer. The TCGA dataset included 497 tumor and 43 normal tissue samples. In the cases where the direction of expression change did not correspond with our methylation change, we visualized complementary TCGA in Illumina 450K DNA methylation array results for the same genes.

### 4.10. Ethics Approval and Consent to Participate

This study was approved by the Ethical Board of the Ruđer Bošković Institute (18 June 2014), the Ethical Board of the Clinical Hospital Dubrava (10 June 2014), and the School of Dental Medicine (10 June 2014), University of Zagreb. The study is in line with the Helsinki Declaration (adopted by the 18th WMA General Assembly, Helsinki, Finland, June 1964; amended by the 29th WMA General Assembly, Tokyo, Japan, October 1975; 35th WMA General Assembly, Venice, Italy, October 1983; 41st WMA General Assembly, Hong Kong, September 1989; 48th WMA General Assembly, Somerset West, Republic of South Africa, October 1996, and the 52nd WMA General Assembly, Edinburgh, Scotland, October 2000) An informed consent to participate in the study was obtained from each participant.

## 5. Conclusions

The presented methylation clustering shows that the potentially premalignant oral lesions (OLL and OLP) are more closely related to healthy mucosa than to the HNSCC although differences between groups exist. The identified panels of hypermethylated and hypomethylated genes, which differentiate the HNSCC samples from oral potentially premalignant lesions and healthy mucosa could clinically be a useful tool for early cancer diagnosis and prognosis. Specific genes that could be considered as HNSCC DNA methylation biomarkers belong to the group of receptor genes, transcription factors, genes involved in adhesion and transport reactions, as well as genes related to the immune response. Thus, the HNSCC hypermethylated *CDH1* gene, involved in cell-cell adhesion, could be considered as a good biomarker for distinguishing cancer tissues from potentially premalignant oral lesions and from healthy oral mucosa. In addition, hypermethylated gene promoters of *SLC5A10*, involved in transport, and *TBC1D2*, involved in cell-cell adhesion, could be also good biomarkers for distinguishing HNSCC from lesions, as well as potentially premalignant oral lesions from healthy oral tissues.

## Figures and Tables

**Table 1:** Hypermethylated gene promoter methylation in HNSCC tissue compared to control healthy tissue and potentially premalignant oral lesions, and in potentially premalignant oral lesions compared to control healthy tissue. The list is merged of top fifteen differentially methylated genes according to the extent of methylation difference value.

Gene Name	Function	MMD *	*p*-Value **
HNSCC Tissue vs. Healthy Tissue			
***GPRC5D***	G protein-coupled receptor	0.78	6.49 × 10^−7^
***TMPRSS11B***	Transmembrane protease	0.76	2.04 × 10^−8^
***PIAS2***	Sumoylation	0.66	1.22 × 10^−6^
***ARG1***	Arginase activity	0.64	2.50 × 10^−7^
***SRPK2***	Protein kinase	0.62	1.30 × 10^−6^
***AADACL2***	Hydrolase activity	0.59	7.38 × 10^−7^
***RGPD4***	RNA transport	0.58	4.71 × 10^−6^
***SPRR3***	Structural molecule activity	0.53	2.44 × 10^−7^
***DEGS1***	Desaturase activity	0.49	2.04 × 10^−8^
***TXNDC8***	Oxidoreductase activity	0.48	1.15 × 10^−5^
***SH3TC1***	Myelination	0.47	2.05 × 10^−6^
***ZPLD1***	Cerebral malformations	0.47	6.17 × 10^−6^
***FBXO2***	Ubiquitination	0.46	0.000236
***ATG16L1***	Autophagy	0.46	0.000306
***GRHL1***	Transcription factor	0.44	5.12 × 10^−7^
**HNSCC Tissue vs. Oral Lesions**			
***RAD51B***	RAD51 Paralog B	0.85	2.53 × 10^−^^8^
***BARX2***	BARX Homeobox 2	0.81	3.94 × 10^−^^8^
***SLC5A10;FAM83G***	Solute Carrier Family 5 Member 10	0.78	2.58 × 10^−^^8^
***NINL***	Ninein Like	0.77	3.96 × 10^−^^8^
***NSMCE2***	NSE2/MMS21 Homolog, SMC5-SMC6 Complex SUMO Ligase	0.76	6.36 × 10^−^^7^
***PGAP2***	Post-GPI Attachment to Proteins 2	0.75	9.48 × 10^−^^8^
***INO80C***	INO80 Complex Subunit C	0.74	1.96 × 10^−^^9^
***IL34***	Interleukin 34	0.74	2.20 × 10^−^^9^
***ZNF516***	Zinc Finger Protein 516	0.73	4.90 × 10^−^^8^
***GFOD2***	Glucose-Fructose Oxidoreductase Domain Containing 2	0.73	1.36 × 10^−^^7^
***PARD3***	Par-3 Family Cell Polarity Regulator	0.73	1.36 × 10^−^^7^
***MCEE***	Methylmalonyl-CoA Epimerase	0.72	2.89 × 10^−^^8^
***POLM***	DNA Polymerase Mu	0.72	3.93 × 10^−^^7^
***ASPG***	Asparaginase	0.71	4.43 × 10^−^^8^
***TBC1D2***	TBC1 Domain Family Member 2	0.71	3.74 × 10^−^^7^
**Oral Lesions vs. Healthy Tissue**			
***SLC5A10;FAM83G***	Solute Carrier Family 5 Member 10	0.78	2.58 × 10^−^^8^
***TBC1D2***	TBC1 Domain Family Member 2	0.71	3.74 × 10^−^^7^
***SH3BP5L***	SH3 Binding Domain Protein 5 Like	0.70	2.68 × 10^−^^7^
***VANGL1***	VANGL Planar Cell Polarity Protein 1	0.69	6.49 × 10^−^^7^
***DLEC1***	Deleted in Lung And Esophageal Cancer 1	0.61	3.84 × 10^−^^6^
***TGOLN2***	Trans-Golgi Network Protein 2	0.61	3.79 × 10^−^^7^
***CTBP2***	C-Terminal Binding Protein 2	0.59	4.99 × 10^−^^6^
***PPP1CB***	Protein Phosphatase 1 Catalytic Subunit Beta	0.56	2.20 × 10^−^^6^
***VPS52***	VPS52, GARP Complex Subunit	0.53	8.05 × 10^−^^6^
***MEPCE***	Methylphosphate Capping Enzyme	0.52	2.89 × 10^−^^7^
***HDAC4***	Histone Deacetylase 4	0.51	6.47 × 10^−^^6^
***ARAP1***	ArfGAP with RhoGAP Domain, Ankyrin Repeat, and PH Domain 1	0.50	4.77 × 10^−^^7^
***TCF20***	Transcription Factor 20	0.49	4.14 × 10^−^^5^
***NDUFS7***	NADH:Ubiquinone Oxidoreductase Core Subunit S7	0.49	0.00017
***GATAD2A***	GATA Zinc Finger Domain Containing 2A	0.47	3.58 × 10^−^^8^

* Mean difference (MD) across all sites in a region (mean.mean.diff); ** false discovery rate (FDR) adjustment combined *p*-value (comb.p.adj.fdr).

**Table 2:** Hypomethylated gene promoter methylation in HNSCC tissue compared to control healthy tissue and potentially premalignant oral lesions, and in potentially premalignant oral lesions compared to control healthy tissue. The list is merged of top fifteen differentially methylated genes according to the extent of methylation difference value.

Gene Name	Function	MMD *	*p*-Value **
HNSCC Tissue vs. Healthy Tissue			
***TRBC2***	T cell receptor	−0.80	2.48 × 10^−7^
***DGAT2***	Acyltransferase activity	−0.70	2.48 × 10^−7^
***ALG1L***	Transferase activity	−0.70	3.29 × 10^−5^
***PDE4D***	Enzyme binding	−0.68	1.06 × 10^−5^
***TRDC***	T cell receptor	−0.67	1.46 × 10^−5^
***DNAJC6***	Phosphatase activity	−0.67	1.63 × 10^−6^
***IGKV3-20***	Immunoglobulin receptor binding	−0.66	1.71 × 10^−5^
***TMEM150B***	Transmembrane protein	−0.66	8.13 × 10^−5^
***LAIR2***	Innate immune response	−0.65	1.81 × 10^−5^
***UBQLN3***	Protein degradation	−0.64	2.57 × 10^−6^
***ANKFN1***	Not known	−0.64	1.71 × 10^−7^
***MS4A1***	Differentiation of B-cells	−0.63	3.80 × 10^−5^
***CCT8L2***	Channel activity	−0.62	3.59 × 10^−6^
***SPOCK1***	Not known	−0.61	5.06 × 10^−5^
***IGHV4-39***	Antigen recognition	−0.61	9.44 × 10^−7^
**HNSCC Tissue vs. Oral Lesions**			
***ART4***	ADP-Ribosyltransferase 4 (Dombrock Blood Group)	−0.88	7.92 × 10^−^^11^
***EPB41L3***	Erythrocyte Membrane Protein Band 4.1 Like 3	−0.87	6.18 × 10^−^^11^
***ESRRG***	Estrogen Related Receptor Gamma	−0.86	8.51 × 10^−^^9^
***ENPP1***	Ectonucleotide Pyrophosphatase/Phosphodiesterase 1	−0.86	1.26 × 10^−^^9^
***GNG7***	G Protein Subunit Gamma 7	−0.86	4.75 × 10^−^^9^
***PAPSS2***	3′-Phosphoadenosine 5′-Phosphosulfate Synthase 2	−0.85	4.10 × 10^−^^9^
***NGEF***	Neuronal Guanine Nucleotide Exchange Factor	−0.84	1.87 × 10^−^^9^
***HIPK4***	Homeodomain Interacting Protein Kinase 4	−0.84	6.69 × 10^−^^9^
***GPR158***	G Protein-Coupled Receptor 158	−0.83	9.82 × 10^−^^10^
***GSG1L***	GSG1 Like	−0.83	1.04 × 10^−^^8^
***SMPD3***	Sphingomyelin Phosphodiesterase 3	−0.83	1.64 × 10^−^^8^
***GDF2***	Growth Differentiation Factor 2	−0.83	5.15 × 10^−^^10^
***RERE***	Arginine-Glutamic Acid Dipeptide Repeats	−0.82	2.19 × 10^−^^8^
***CDH13***	Cadherin 13	−0.82	1.81 × 10^−^^10^
***HS3ST4***	Heparan Sulfate-Glucosamine 3-Sulfotransferase 4	−0.82	1.02 × 10^−^^8^
**Oral Lesions vs. Healthy Tissue**			
***ART4***	ADP-Ribosyltransferase 4 (Dombrock Blood Group)	−0.88	7.92 × 10^−11^
***ENPP1***	Ectonucleotide Pyrophosphatase/Phosphodiesterase 1	−0.86	1.26 × 10^−9^
***GNG7***	G Protein Subunit Gamma 7	−0.86	4.75 × 10^−9^
***PKD1L3***	Polycystin 1 Like 3, Transient Receptor Potential Channel Interacting	−0.81	2.20 × 10^−9^
***PLXNC1***	Plexin C1	−0.81	1.41 × 10^−9^
***CAMK2B***	Calcium/Calmodulin Dependent Protein Kinase II Beta	−0.79	1.05 × 10^−8^
***CACNA1S***	Calcium Voltage–Gated Channel Subunit Alpha1 S	−0.78	8.18 × 10^−9^
***SCGB1D1***	Secretoglobin Family 1D Member 1	−0.78	2.34 × 10^−7^
***VPS13D***	Vacuolar Protein Sorting 13 Homolog D	−0.76	6.12 × 10^−8^
***DLGAP4***	DLG Associated Protein 4	−0.76	6.37 × 10^−9^
***LRP1B***	LDL Receptor Related Protein 1B	−0.76	1.12 × 10^−9^
***COL2A1***	Collagen Type II Alpha 1 Chain	−0.75	1.30 × 10^−8^
***SLC24A3***	Solute Carrier Family 24 Member 3	−0.74	3.07 × 10^−9^
***TBC1D8***	TBC1 Domain Family Member 8	−0.74	1.84 × 10^−8^
***ABCC8***	ATP Binding Cassette Subfamily C Member 8	−0.73	3.58 × 10^−9^

* Mean difference (MD) across all sites in a region (mean.mean.diff); ** false discovery rate (FDR) adjustment combined *p*-value (comb.p.adj.fdr).

**Table 3:** Hypermethylated 5′-cytosine-phosphate-guanine-3′ (CpG) sites methylation in HNSCC tissue compared to control healthy tissue and potentially premalignant oral lesions, and in potentially premalignant oral lesions compared to control healthy tissue. The list is merged of top fifteen differentially methylated genes according to the extent of methylation difference value.

Gene Name	Function	cg Position	MMD *	*p*-Value **
HNSCC Tissue vs. Healthy Tissue				
***LMBR1L***	Probable receptor	cg12348519	0.93	4.59 × 10^−8^
***CDH1***	Adhesions, mobility, and proliferation	cg08285862	0.92	3.49 × 10^−8^
***EIF6***	Initiation of translation	cg09957666	0.92	1.96 × 10^−8^
***C16orf70***	Not known	cg03664901	0.92	3.48 × 10^−8^
***ETNK2***	Transferase and kinase activity	cg12142497	0.92	5.33 × 10^−8^
***C11orf73***	Cellular response to heat stress	cg23450586	0.91	5.01 × 10^−9^
***ADARB2***	RNA editing	cg26569590	0.91	2.10 × 10^−8^
***GAB1***	Cellular growth, transformation, and apoptosis	cg23020414	0.91	2.83 × 10^−8^
***ITPR3***	Metabolism and growth	cg05876496	0.91	6.35 × 10^−8^
***WDR61***	Transcriptional regulation	cg12339790	0.90	4.35 × 10^−8^
***PGAP2***	Protein transport	cg01156876	0.90	1.24 × 10^−8^
***DDX10***	RNA helicase	cg18585558	0.90	6.14 × 10^−9^
***DGKH***	Kinase activity	cg22899750	0.90	1.09 × 10^−7^
***RAB40C***	Oncogene	cg01770948	0.89	2.00 × 10^−8^
***BEAN1***	Not known	cg19471156	0.89	5.59 × 10^−8^
**HNSCC Tissue vs. Oral Lesions**				
***EIF6***	Eukaryotic Translation Initiation Factor 6	cg09957666	0.91	5.68 × 10^−^^10^
***KANSL1***	KAT8 Regulatory NSL Complex Subunit 1	cg07281649	0.91	1.43 × 10^−^^9^
***DDX10***	DEAD–Box Helicase 10	cg18585558	0.89	3.58 × 10^−^^10^
***AP2A1***	Adaptor Related Protein Complex 2 Alpha 1 Subunit	cg08969148	0.89	8.97 × 10^−^^10^
***RAB40C***	RAB40C, Member RAS Oncogene Family	cg01770948	0.89	1.84 × 10^−^^9^
***GAB1***	GRB2 Associated Binding Protein 1	cg23020414	0.88	5.30 × 10^−^^9^
***ERGIC1***	Endoplasmic Reticulum-Golgi Intermediate Compartment 1	cg07769006	0.88	1.25 × 10^−^^9^
***SNX14***	Sorting Nexin 14	cg03776905	0.88	3.20 × 10^−^^9^
***PIGU***	Phosphatidylinositol Glycan Anchor Biosynthesis Class U	cg09450087	0.88	1.22 × 10^−^^10^
***ARAP1***	ArfGAP with RhoGAP Domain, Ankyrin Repeat, and PH Domain 1	cg09010791	0.87	1.56 × 10^−^^9^
***LMTK2***	Lemur Tyrosine Kinase 2	cg05941925	0.87	2.59 × 10^−^^9^
***BEAN1***	Brain Expressed Associated with NEDD4 1	cg19471156	0.87	6.96 × 10^−^^9^
***AP1S3***	Adaptor Related Protein Complex 1 Sigma 3 Subunit	cg25666945	0.87	1.66 × 10^−^^9^
***CDH1***	Cadherin 1	cg08285862	0.87	2.28 × 10^−^^8^
***RYBP***	RING1 and YY1 Binding Protein	cg08086385	0.86	3.11 × 10^−^^10^
**Oral Lesions vs. Healthy Tissue**				
***GRIP1***	Glutamate Receptor Interacting Protein 1	cg09414535	0.68	0.000679
***MTMR10***	Myotubularin Related Protein 10	cg25430175	0.66	0.000585
***RBM47***	RNA Binding Motif Protein 47	cg11268702	0.66	0.000636
***MPHOSPH9***	M–Phase Phosphoprotein 9	cg02132191	0.65	0.001055
***FOXK1***	Forkhead Box K1	cg16026475	0.64	0.000765
***SNX3***	Sorting Nexin 3	cg14452952	0.64	0.000825
***CIT***	Citron Rho-Interacting Serine/Threonine Kinase	cg03601895	0.63	0.000866
***ZBTB38***	Zinc Finger and BTB Domain Containing 38	cg13318410	0.63	0.001548
***DRD3***	Dopamine Receptor D3	cg22253817	0.63	0.001115
***SPPL3***	Signal Peptide Peptidase Like 3	cg11330512	0.63	0.001072
***ZNF407***	Zinc Finger Protein 407	cg23863184	0.63	0.000942
***ADAMTSL1***	ADAMTS Like 1	cg12699984	0.62	0.000767
***GNAT3***	G Protein Subunit Alpha Transducin 3	cg10168361	0.62	0.000936
***L3MBTL3***	L3MBTL3, Histone Methyl-Lysine Binding Protein	cg22162357	0.62	0.001083
***EEPD1***	Endonuclease/Exonuclease/Phosphatase Family Domain Containing 1	cg06387870	0.61	0.001083

* Mean difference (MD) across all sites in a region (mean.mean.diff); ** false discovery rate (FDR) adjustment combined *p*-value (comb.p.adj.fdr).

**Table 4:** Hypomethylated CpG sites methylation in HNSCC tissue compared to control healthy tissue and potentially premalignant oral lesions, and in potentially premalignant oral lesions compared to control healthy tissue. The list is merged of top fifteen differentially methylated genes according to the extent of methylation difference value.

Gene Name	Function	cg Position	MMD *	*p*-Value **
HNSCC Tissue vs. Healthy Tissue				
***ATXN1***	Not known	cg07713291	−0.96	2.97 × 10^−9^
***PPP2R2C***	Cell growth	cg05805165	−0.93	1.22 × 10^−8^
***CCR6***	Immune regulation	cg05094429	−0.92	1.08 × 10^−7^
***RAB37***	Oncogene	cg25267982	−0.92	1.29 × 10^−8^
***DUSP27***	Phosphatase activity	cg23713934	−0.91	1.94 × 10^−7^
***ZNF521***	Transcription factor	cg21830945	−0.91	7.58 × 10^−8^
***SLC6A17***	Transporter	cg12072789	−0.90	7.38 × 10^−8^
***SPIN1***	Methylated histone binding	cg13554018	−0.90	1.63 × 10^−8^
***CXCR1***	Receptor	cg13519373	−0.90	1.22 × 10^−8^
***SPTBN1***	Cell shape	cg06149826	−0.89	6.91 × 10^−9^
***NBAS***	Golgi to ER transport	cg27424261	−0.89	5.31 × 10^−7^
***NRG3***	Ligand	cg10656958	−0.89	9.45 × 10^−8^
***COL5A1***	Forming collagen	cg26087052	−0.89	3.96 × 10^−8^
***CDX1***	Transcriptional regulation	cg12473781	−0.89	5.77 × 10^−8^
***BATF3***	Transcriptional regulation	cg03219362	−0.89	2.10 × 10^−8^
**HNSCC Tissue vs. Oral Lesions**				
***FAM69A***	Family with Sequence Similarity 69 Member A	cg22727960	−0.93	7.05 × 10^−11^
***ATP6V0A1***	ATPase H+ Transporting V0 Subunit A1	cg19022525	−0.92	9.01 × 10^−11^
***LBP***	Lipopolysaccharide Binding Protein	cg18979491	−0.92	3.02 × 10^−10^
***WDR25***	WD Repeat Domain 25	cg24211276	−0.91	6.22 × 10^−11^
***SH3RF3***	SH3 Domain Containing Ring Finger 3	cg27294813	−0.91	1.01 × 10^−9^
***NINJ2***	Ninjurin 2	cg05534515	−0.91	2.74 × 10^−12^
***RAB37***	RAB37, Member RAS Oncogene Family	cg25267982	−0.90	1.17 × 10^−9^
***CXCR1***	C-X-C Motif Chemokine Receptor 1	cg13519373	−0.90	1.76 × 10^−10^
***SPTBN***	Spectrin Beta, Non–Erythrocytic 1	cg06149826	−0.90	1.54 × 10^−10^
***RHOH***	Ras Homolog Family Member H	cg15729055	−0.90	1.90 × 10^−9^
***GRIK5***	Glutamate Ionotropic Receptor Kainate Type Subunit 5	cg03100024	−0.90	2.47 × 10^−9^
***KLRD1***	Killer Cell Lectin Like Receptor D1	cg05377120	−0.90	6.88 × 10^−9^
***TENM2***	Teneurin Transmembrane Protein 2	cg26758826	−0.89	3.56 × 10^−11^
***FAM69A***	Family with Sequence Similarity 69 Member A	cg05172999	−0.89	3.14 × 10^−10^
***ITK***	IL2 Inducible T Cell Kinase	cg12250498	−0.89	3.46 × 10^−10^
**Oral Lesions vs. Healthy Tissue**				
***PHACTR1***	Phosphatase and Actin Regulator 1	cg02381687	−0.80	0.000673
***MARCH8***	Membrane Associated Ring-CH-Type Finger 8	cg26841425	−0.80	0.000585
***PPP1R1B***	Protein Phosphatase 1 Regulatory Inhibitor Subunit 1B	cg03104421	−0.79	0.000585
***HDAC4***	Histone Deacetylase 4	cg21190228	−0.79	0.00057
***IL22RA2***	Interleukin 22 Receptor Subunit Alpha 2	cg23507945	−0.79	0.001772
***CAMKK2***	Calcium/Calmodulin Dependent Protein Kinase Kinase 2	cg03391567	−0.78	0.000679
***INPP5D***	Inositol Polyphosphate-5-Phosphatase D	cg22666015	−0.78	0.000709
***CSGALNACT1***	Chondroitin Sulfate N–Acetylgalactosaminyltransferase 1	cg24423468	−0.77	0.001266
***GTDC1***	Glycosyltransferase Like Domain Containing 1	cg19251811	−0.77	0.000676
***IGSF3***	Immunoglobulin Superfamily Member 3	cg13004173	−0.77	0.000585
***HELZ***	Helicase with Zinc Finger	cg15015109	−0.76	0.000772
***DEFA4***	Defensin Alpha 4	cg06617936	−0.76	0.000678
***AK5***	Adenylate Kinase 5	cg21487631	−0.76	0.000681
***LHFPL2***	LHFPL Tetraspan Subfamily Member 2	cg20879720	−0.76	0.000981
***STK10***	Serine/Threonine Kinase 10	cg22406187	−0.76	0.000765

* Mean difference (MD) across all sites in a region (mean.mean.diff); ** false discovery rate (FDR) adjustment combined *p*-value (comb.p.adj.fdr).

## Supplementary Materials

Supplementary materials can be found at https://www.mdpi.com/1422-0067/21/18/6853/s1.

## Abbreviations

comb.p.adj.fdr	FDR adjusted combined *p*-value;
CpG	5′-cytosine-phosphate-guanine-3′
FDR	false discovery rate
GSEA	gene set enrichment analysis
HNSCC	head and neck squamous cell carcinoma
HPV	human papillomavirus
KEGG	Kyoto encyclopedia of genes and genomes
limma	linear models for microarray data
mean.mean.diff	value of the mean difference across all sites in a region
miRNA	micro ribonucleic acids
NA	not annotated; NTA, network topology-based analysis
OLL	oral lichenoid lesions
OLP	oral lichen planus
OPSCC	oropharyngeal squamous cell carcinoma
ORA	over-representation enrichment analysis
OSCC	oral squamous cell carcinoma
PCA	principal component analysis
PCR	polymerase chain reaction
RnBeads	integrated software package for the analysis and interpretation of DNA methylation data
SNPs	single nucleotide polymorphisms
TCGA	The Cancer Genome Atlas
TS	type-specific
