# Supplementary material for: DNA Methylome Distinguishes Head and Neck Cancer from Potentially Malignant Oral Lesions and Healthy Oral Mucosa
Source: Int J Mol Sci. 2020 Sep 18;21(18):6853. doi: 10.3390/ijms21186853 (PMC7554960; doi:10.3390/ijms21186853)
Supplement: Supplementary file 1 [file ijms-21-06853-s001.pdf]

Supplementary

Table S1. Characteristics of pyrosequencing primers.

Primer ID	Primer sequence	Ta (°C)	Amplicon size
TRDC F	GAATGATTTAGGAGGTAGAGTTTGT		
TRDC R*	ACCTCCAATCACTTCAAACTTCAT	63 °C	132 bp
TRDC S	AGGAGGTAGAGTTTGTA		
SPRR3-1 F	TAGTGTATTGTTTGGAAGGTAGT		
SPRR3-1 R*	CCATTCAACTACTTCTTCCTACT	57 °C	286 bp
SPRR3-1 S	ATAATTGGTTTTTTGATTTTTTTAA		
SPRR3-2 S	TTTTTATATATAGGGAAATATTG		
LAIR2-1 F	TGTGGTTTTGGTTTTTGTGTAAG		
LAIR2-1 R*	CTTCAATCAAACCCAAAATTCATCCT	57 °C	194 bp
LAIR2-1 S	TGGTTTTTGTGTAAGAGT		
LAIR2-2 S	TGGGGTTTGAGAGAT		
FBXO2 F	AGATGGGTATGGTGGTATTTG		
FBXO2 R*	CTAACCTCCAATACCCACTTCTATC	55 °C	253 bp
FBXO2 S	GGTGGTATTTGTTTGTAAT		

* 5′ biotinylated primer

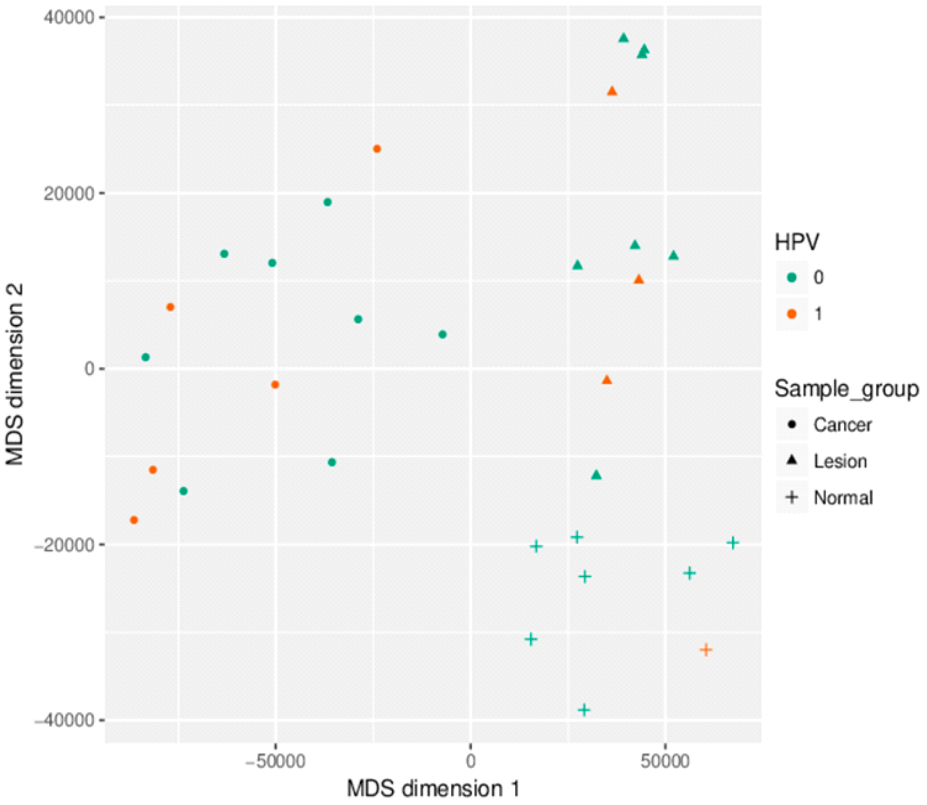

Figure S1. Infinium MethylationEPIC BeadChip findings. HPV positivity (orange) displayed across sample groups.

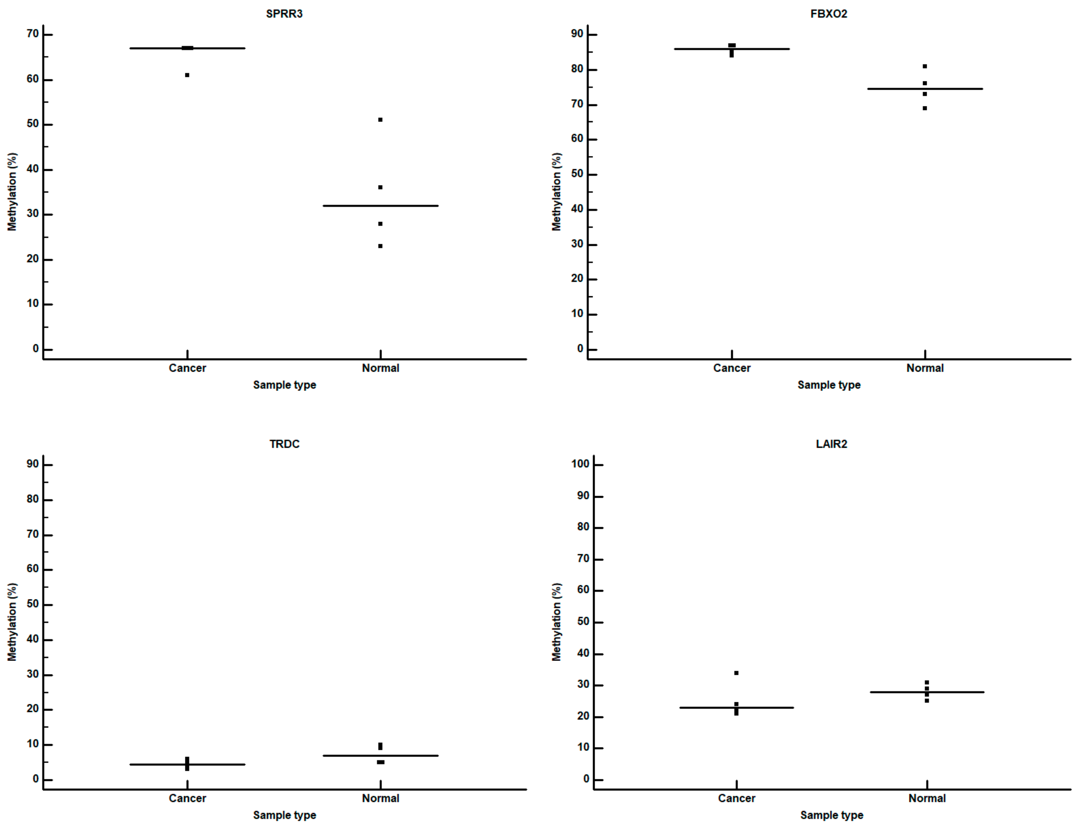

Figure S2. Pyrosequencing validation of methylated gene promoters: *SPRR3*, *FBXO2*, *TRDC*, and *LAIR2*. The statistical significance was reached between HNSCC and the control samples in only CpG1 and CpG3 of *SPRR3* gene ($p = 0.01$ in both cases) and CpG1 of *FBXO2* gene ($p = 0.01$).

External database validation

Our methylation findings were compared to TCGA Illumina HISeq RNAseq data of the TCGA-HNSC project through Wanderer (http://maplab.imppc.org/wanderer/), an interactive viewer to explore DNA methylation and gene expression data in human cancer. The RNAseq estimate of expression for the top 15 hypo- and hypermethylated gene promoters in our results were visualized in Wanderer. The TCGA dataset included 497 tumor and 43 normal tissue samples. Where the direction of expression change did not correspond with our methylation change, we also visualized a complementary TCGA in Illumina 450K DNA methylation array results for the same genes. The summary table (**Table A2**) and box plot graphs for individual genes are provided below. Out of the total of top 15 hypermethylated genes in our study, 10 were found to be either under-expressed or hypermethylated in TCGA cancer cases, as expected, while one had no measurable expression and only a single CpG site in Illumina 450K DNA methylation array. From the top 15 hypomethlylated genes in our study, 12 were also found to be either over-expressed or hypomethylated in TCGA data, with the remaining three lacking annotated data or probes in Illumina 450K DNA methylation array (Díez-Villanueva, Anna, Izaskun Mallona, and Miguel A. Peinado. "Wanderer, an Interactive Viewer to Explore DNA Methylation and Gene Expression Data in Human Cancer." *Epigenetics and Chromatin* 8, no. 1 (June 23, 2015): 22 https://doi.org/10.1186/s13072-015-0014-8).

Table S2. Comparison of our methylation data with previous TCGA expression data for the same genes. Where the expression change was discrepant, TCGA methylation data was assessed. The bold font indicates the expected results or confirmed genes, italic fonts indicate discrepancies.

Methylation	Gene promoters	TCGA expression in cancer	TCGA methylation in cancer

Hypermethylated in cancer in our study	GPRC5D	*no difference*	
	**TMPRSS11B**	**underexpressed**	
	**PIAS2**	**underexpressed**	
	**ARG1**	**underexpressed**	
	**SRPK2**	*overexpressed*	**some sites hypermethylated**
	**AADACL2**	**underexpressed**	
	**RGPD4**	**underexpressed**	
	**SPRR3**	**underexpressed**	
	**DEGS1**	*overexpressed*	**some sites hypermethylated**
	TXNDC8	*not expressed*	no difference, single site
	**SH3TC1**	*overexpressed*	**closest CpG site to start hypermethylated**
	ZPLD1	*overexpressed*	mostly hypomethylated
	FBXO2	*overexpressed*	mostly hypomethylated
	ATG16L1	*no difference*	mostly hypomethylated
	**GRHL1**	**underexpressed**	
Hypomethylated in cancer in our study	**TRBC2**	**overexpressed**	
	**DGAT2**	*underexpressed*	**most sites hypomethylated**
	**ALG1L**	**overexpressed**	
	**PDE4D**	*underexpressed*	**most sites hypomethylated**
	TRDC	*no difference*	no probes in 450k
	**DNAJC6**	**overexpressed**	
	IGKV3-20	not annotated	not annotated
	**TMEM150B**	**overexpressed**	
	**LAIR2**	**overexpressed**	
	**UBQLN3**	*no difference*	**strongly hypomethylated**
	**ANKFN1**	*underexpressed*	**strongly hypomethylated**
	**MS4A1**	*no difference*	**strongly hypomethylated**
	**CCT8L2**	*not annotated*	**strongly hypomethylated**
	**SPOCK1**	**overexpressed**	
	IGHV4-39	not annotated	not annotated

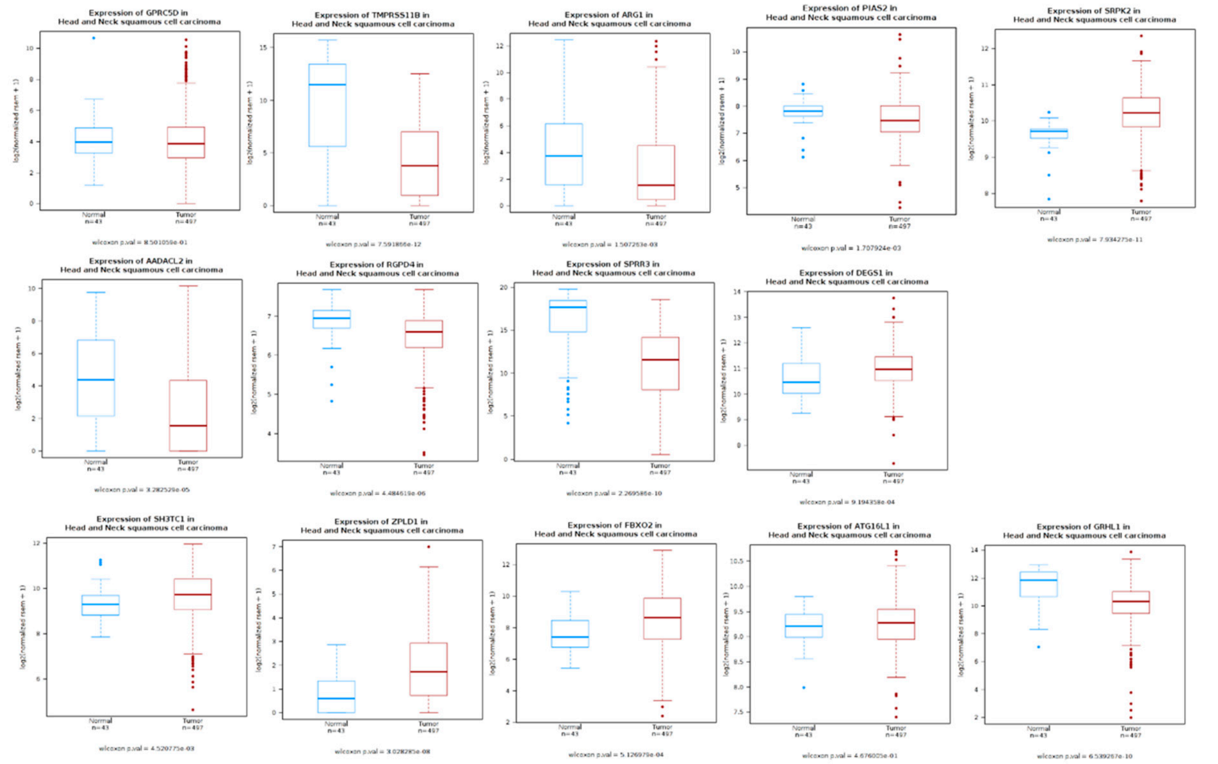

Figure S3. Public TCGA expression data of the selected top 15 genes found to be hypermethylated in our study. Graphs were interactively made in Wanderer web server from the TCGA-HNSCC project from Illumina HiSeq RNAseq data.

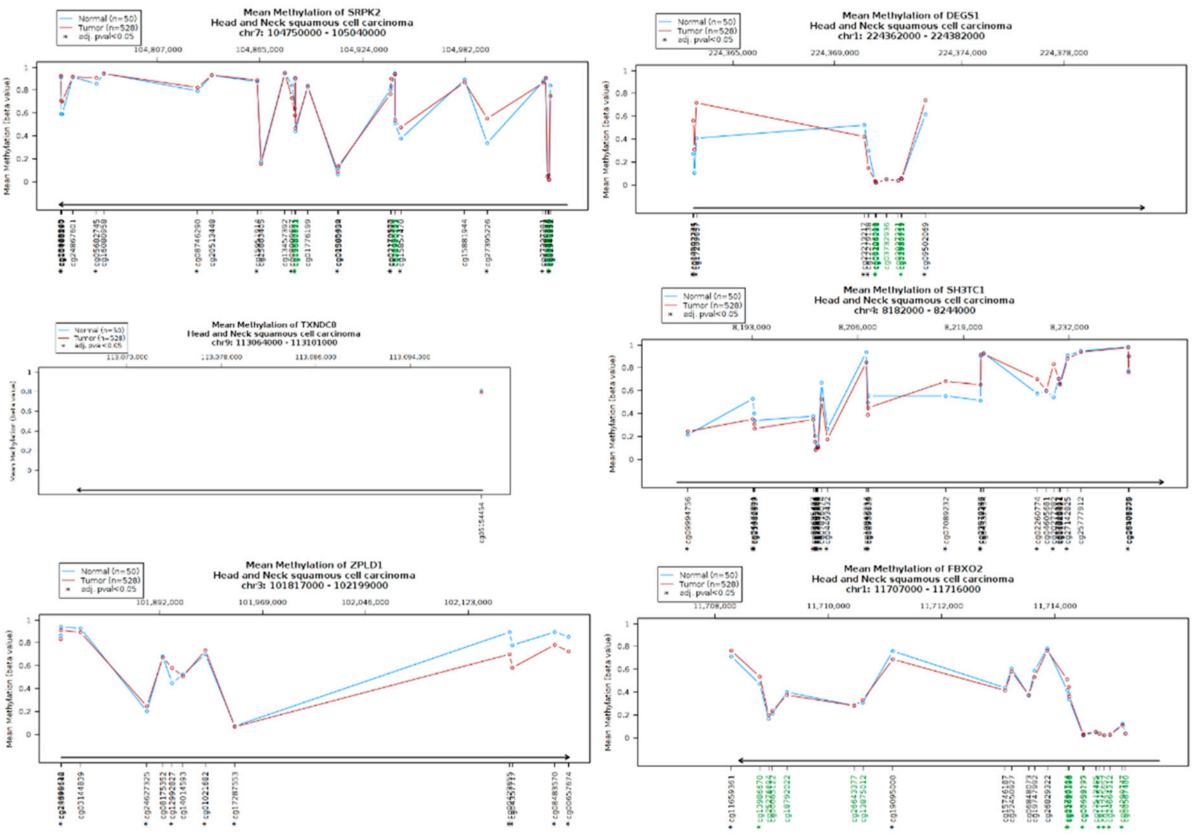

Figure S4. Public TCGA methylation data of genes found to be hypermethylated in our study but without decreased expression in TCGA RNAseq data. Graphs were interactively made in Wanderer web server from the TCGA-HNSCC project from Illumina 450K DNA methylation array.

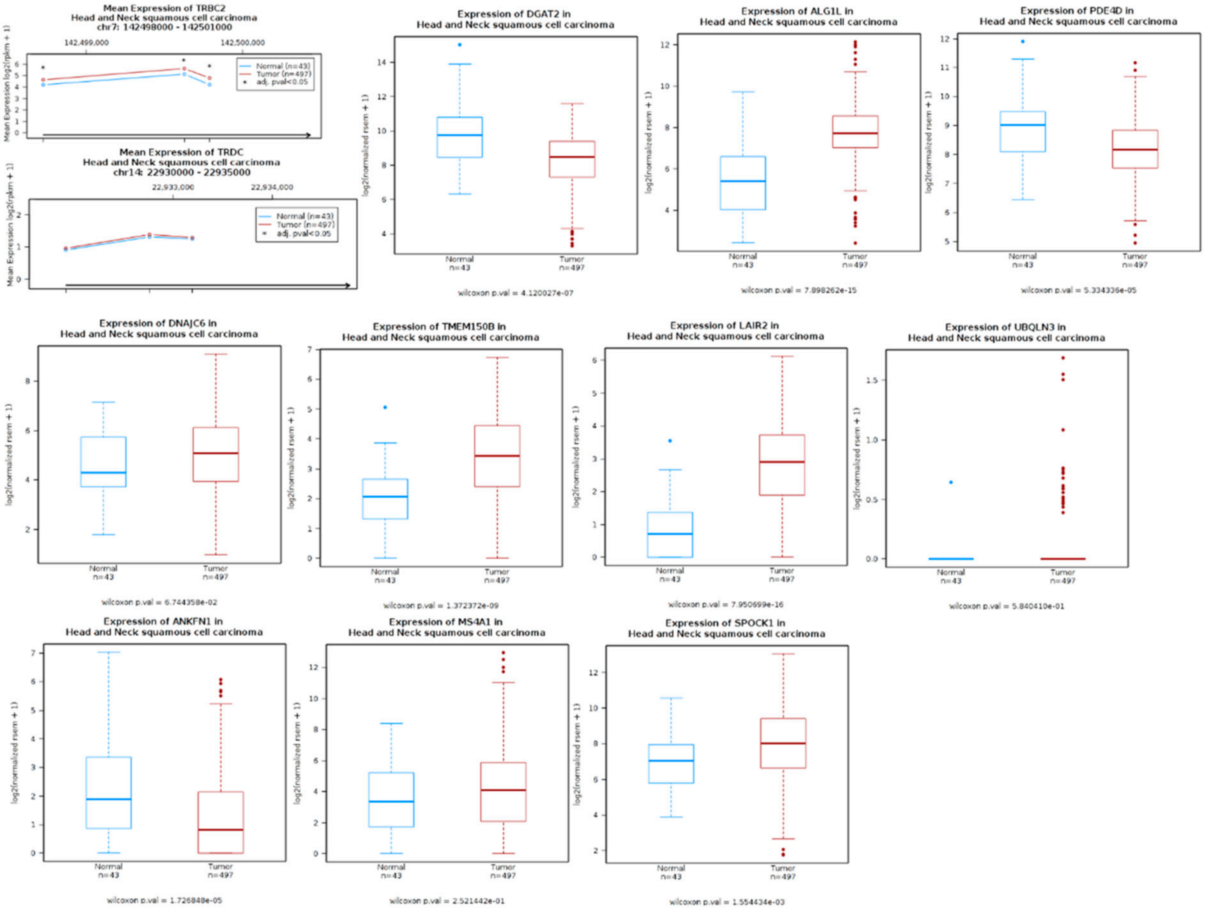

Figure S5. Public TCGA expression data of the selected top 15 genes found to be hypomethylated in our study. Graphs were interactively made in Wanderer web server from the TCGA-HNSCC project from Illumina HiSeq RNAseq data.

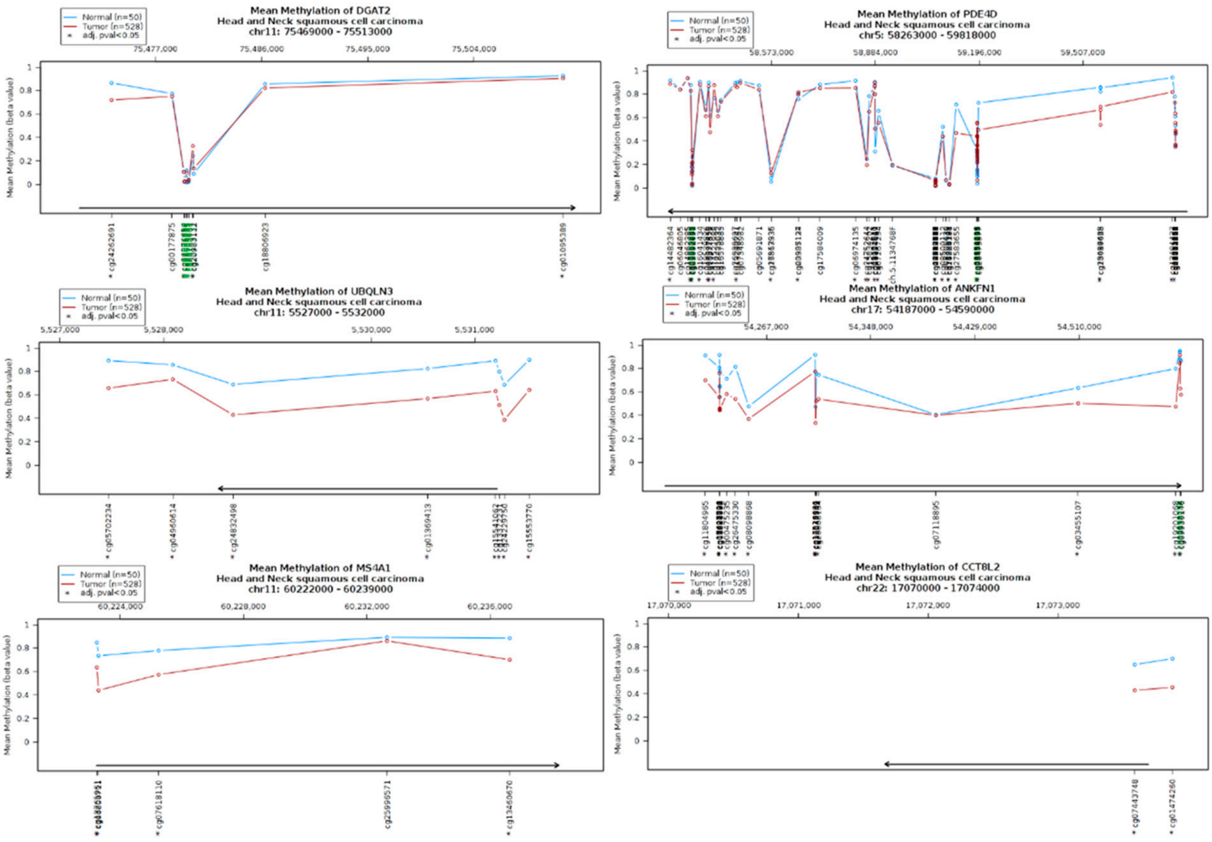

Figure S6. Public TCGA methylation data of genes found to be hypomethylated in our study but without increased expression in TCGA RNAseq data. Graphs were interactively made in Wanderer web server from the TCGA-HNSCC project from Illumina 450K DNA methylation array.

Gene set enrichment analysis

The list of differentially methylated CpG and promotor regions was combined and assessed to determine whether the affected genes are enriched for specific sets of functions or pathways. However, for the analysis, only those sites/regions with assigned RefGene names indicating nearby or overlapping genes were selected. The analysis was done using the WebGestalt functional enrichment analysis web tool (Liao, Yuxing, Jing Wang, Eric J. Jaehnig, Zhiao Shi, and Bing Zhang. "WebGestalt 2019: Gene Set Analysis Toolkit with Revamped UIs and APIs." Nucleic Acids Research. Accessed 28 May 2019. https://doi.org/10.1093/nar/gkz401).

Methylation data was separately explored for the hypo- and hypermethylated genes of three comparisons: HNSCC vs. healthy tissue, HNSCC vs. oral lesion, and oral lesion vs. normal healthy tissue. Two different analysis approaches were used: Over Representation Enrichment Analysis (ORA) and Gene Set Enrichment Analysis (GSEA). For the ORA and GSEA analysis, the Gene Ontology (GO) of biological process (no-redundant) and Kyoto Encyclopedia of Genes and Genomes (KEGG) pathway databases were chosen.

For ORA, the reference gene set was set to the whole genome, since many differentially methylated regions were related to miRNA and other non-coding sequences. For GSEA, gene promotors were ranked according to the mean difference and this data was supplied in addition to the gene symbol. Unless indicated otherwise, default parameters were used. The results are presented below. The ORA (GO biological processes) for consistantly hypomethylated gene promoters and/or CpG sites in A) HNSCC compared to control healthy tissue, B) HNSCC compared to potentially premalignant oral lesions, and C) potentially premalignant oral lesions compared to control healthy tissue is shown in

Figure 4 in the main publication; the top 10 categories and False Discovery Rate (FDR) adjusted significance (colored bar) are shown.

A – HNSCC tissue vs. healthy oral tissue.

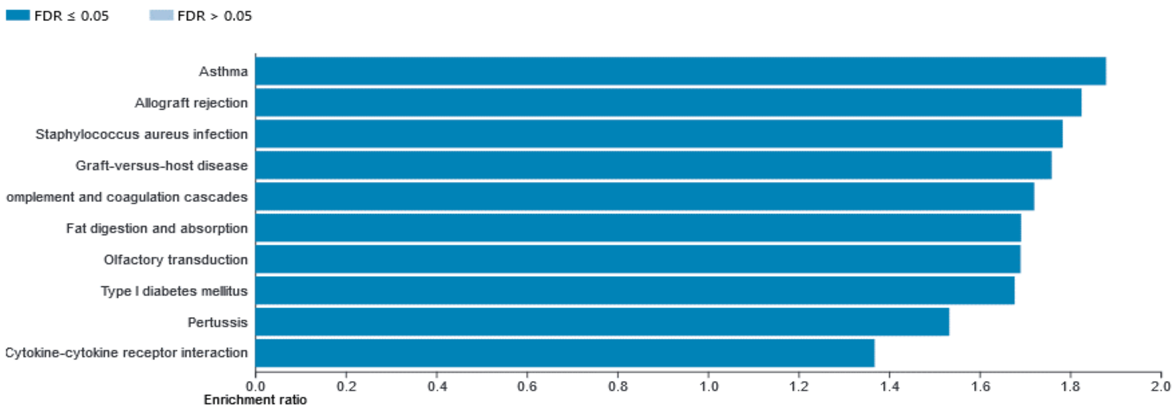

B – HNSCC tissue vs. oral lesions.

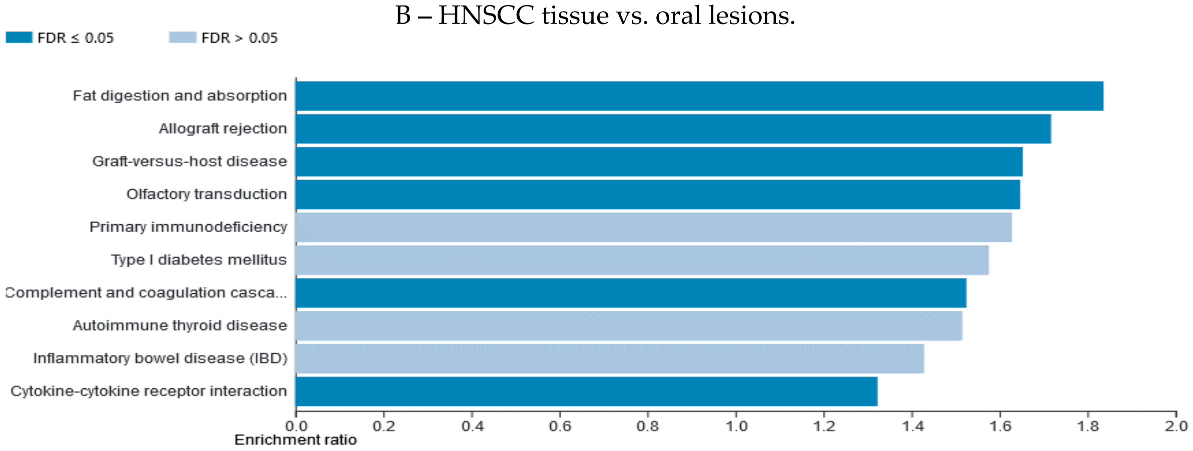

C – Oral lesions vs. healthy oral tissue.

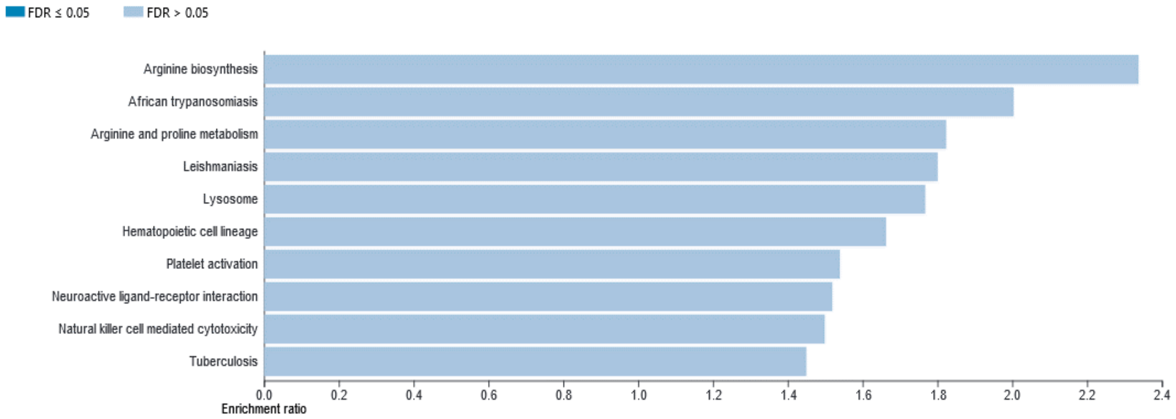

Figure S7. ORA analysis of KEGG pathway for hypomethylated gene promoters and/or CpG sites in A) HNSCC tissue compared to healthy oral tissue, B) HNSCC tissue compared to potentially premalignant oral lesions, and C) potentially premalignant oral lesions compared to healthy oral tissue. The top 10 categories are shown; FDR adjusted significance is indicated as the colored bar.

A – HNSCC tissue vs. healthy oral tissue.

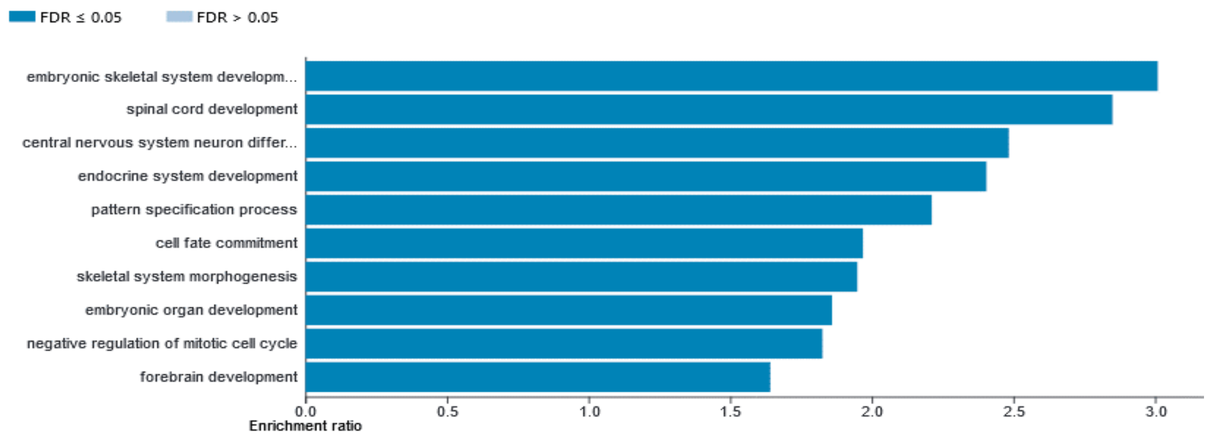

B – HNSCC tissue vs. oral lesions.

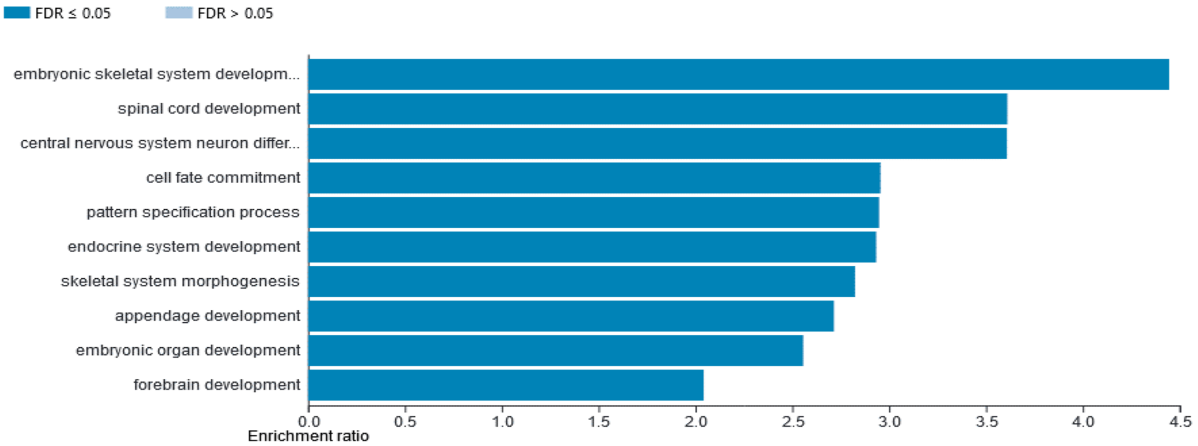

C – Oral lesions vs. healthy oral tissue.

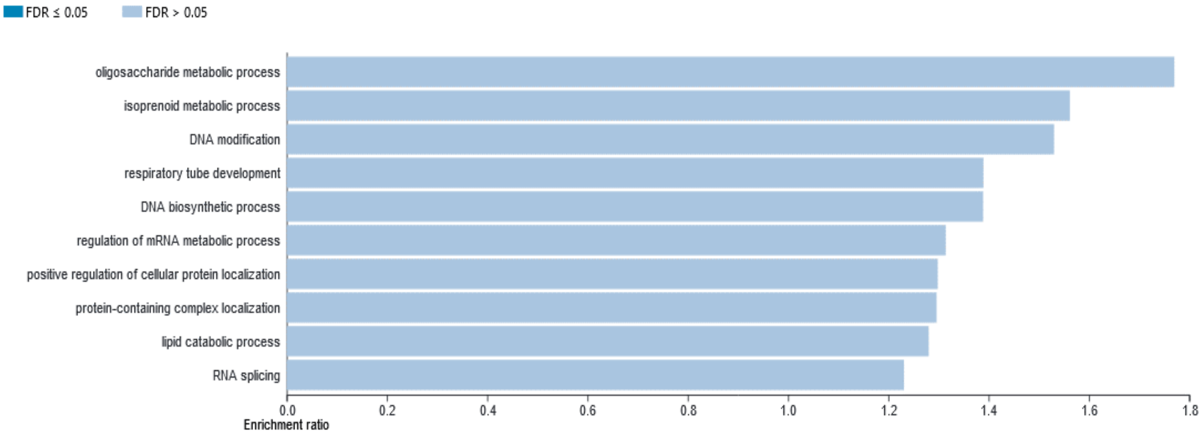

Figure S8. ORA analysis of GO biological processes for hypermethylated gene promoters and/or CpG sites in A) HNSCC tissue compared to healthy oral tissue, B) HNSCC tissue compared to potentially premalignant oral lesions, and C) potentially premalignant oral lesions compared to healthy oral tissue. The top 10 categories are shown; FDR adjusted significance is indicated as the colored bar.

A – HNSCC tissue vs. healthy oral tissue.

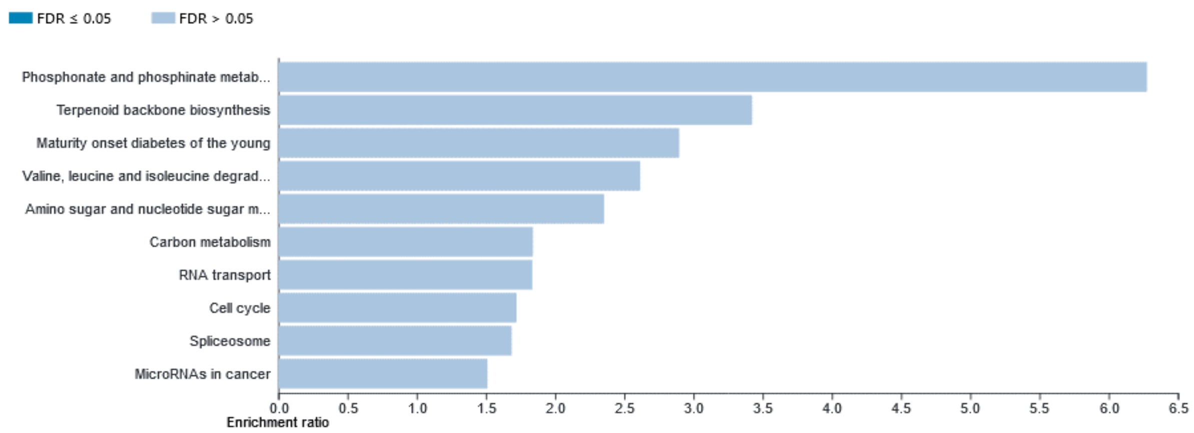

B – HNSCC tissue vs. oral lesions.

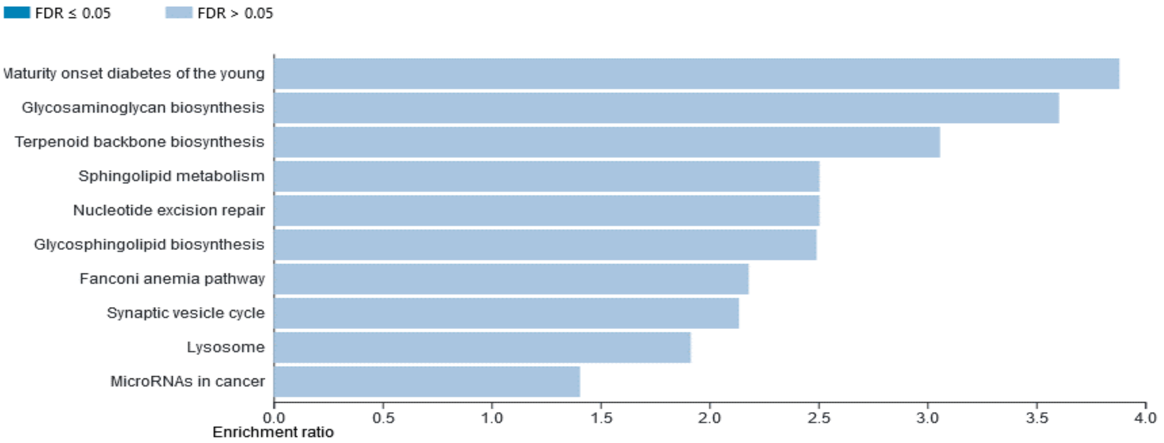

C – Oral lesions vs. healthy oral tissue.

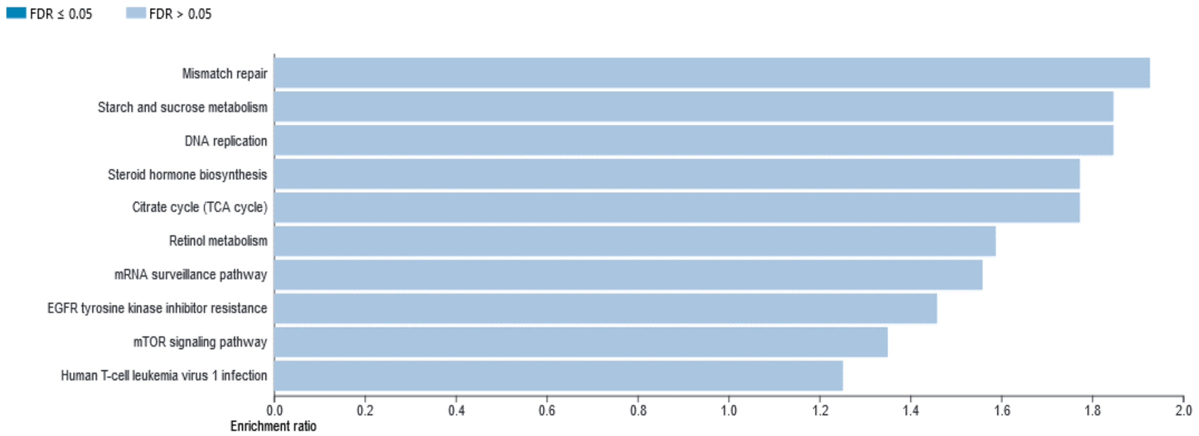

Figure S9. ORA analysis of KEGG pathway for hypermethylated gene promoters and/or CpG sites in A) HNSCC tissue compared to healthy oral tissue, B) HNSCC tissue compared to potentially premalignant oral lesions, and C) potentially premalignant oral lesions compared to healthy oral tissue. The top 10 categories are shown; FDR adjusted significance is indicated as the colored bar.

A – HNSCC tissue vs. healthy oral tissue.

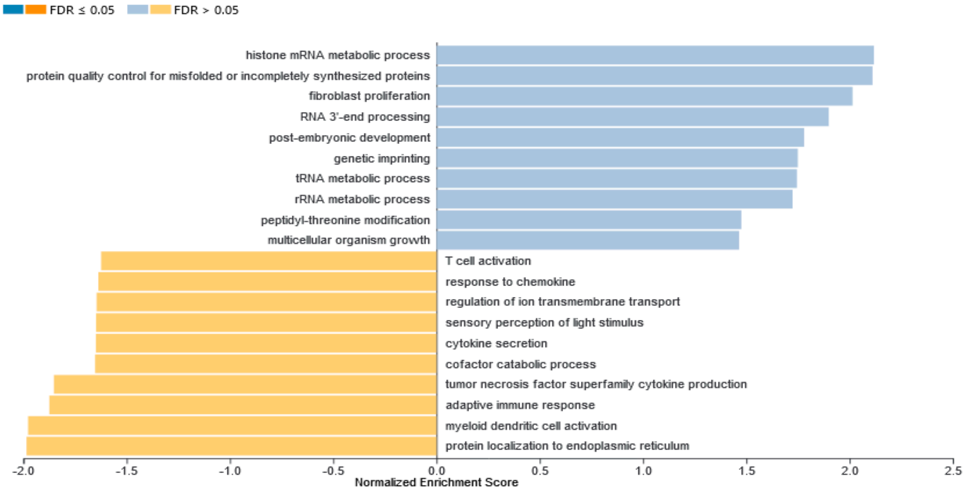

B – HNSCC tissue vs. oral lesions.

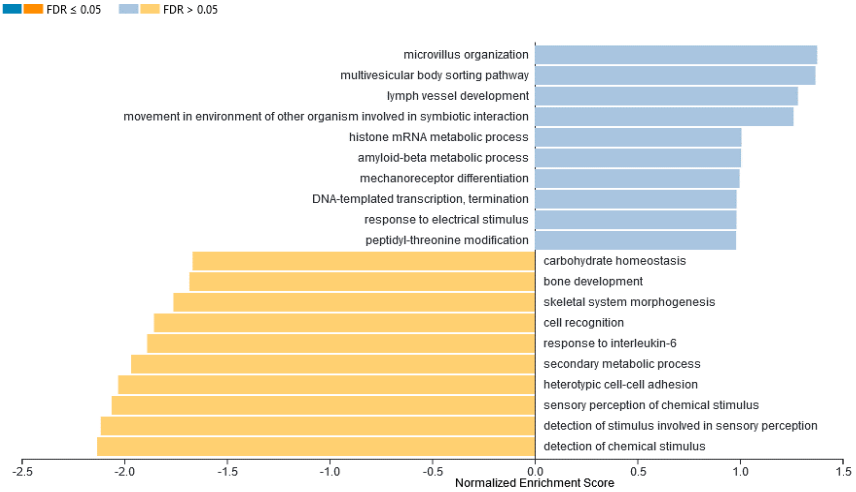

C – Oral lesions vs. healthy oral tissue.

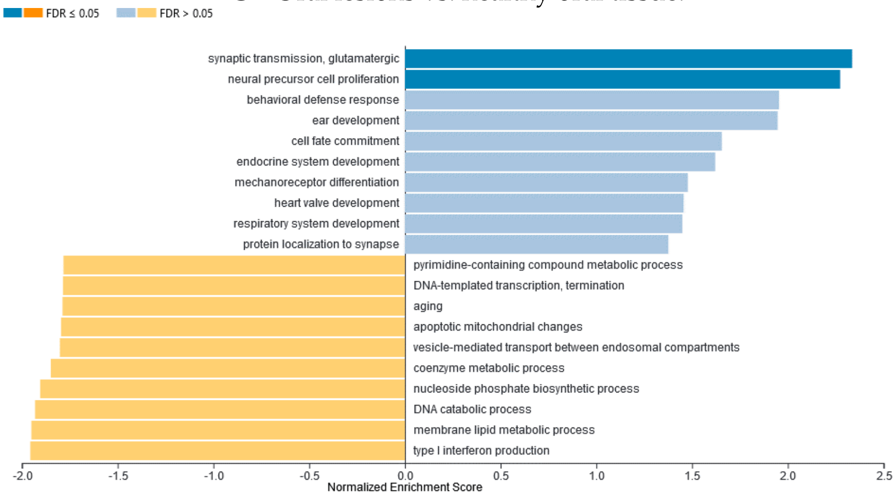

Figure S10. GSEA analysis of GO biological processes for hypomethylated gene promoters and/or CpG sites in (**A**) HNSCC tissue compared to healthy oral tissue, (**B**) HNSCC tissue compared to potentially premalignant oral lesions, and (**C**) potentially premalignant oral lesions compared to healthy oral tissue. The top 10 categories are shown; FDR adjusted significance is indicated as the colored bar.

A – HNSCC tissue vs. healthy oral tissue.

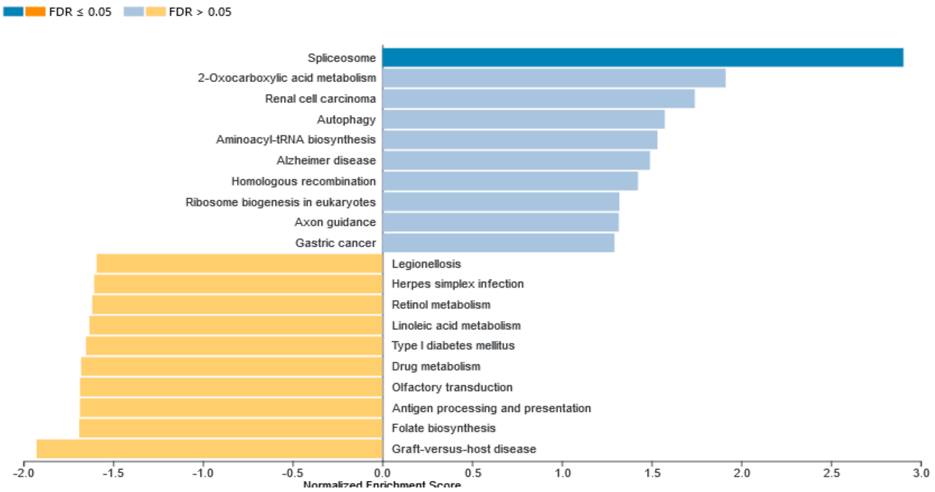

B – HNSCC tissue vs. oral lesions.

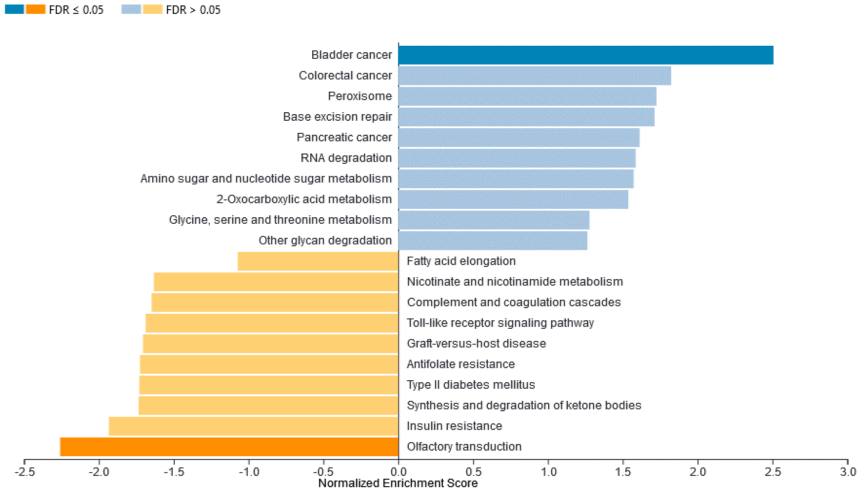

C – Oral lesions vs. healthy oral tissue.

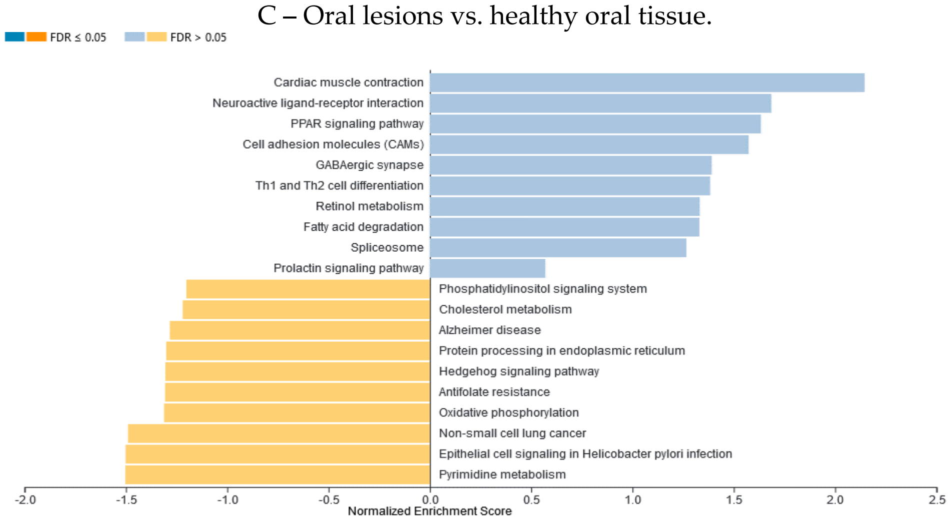

Figure S11. GSEA analysis of KEGG pathway for hypomethylated gene promoters and/or CpG sites in (**A**) HNSCC tissue compared to healthy oral tissue, (**B**) HNSCC tissue compared to potentially premalignant oral lesions, and (**C**) potentially premalignant oral lesions compared to healthy oral tissue. The top 10 categories are shown; FDR adjusted significance is indicated as the colored bar.

A – HNSCC tissue vs. healthy oral tissue.

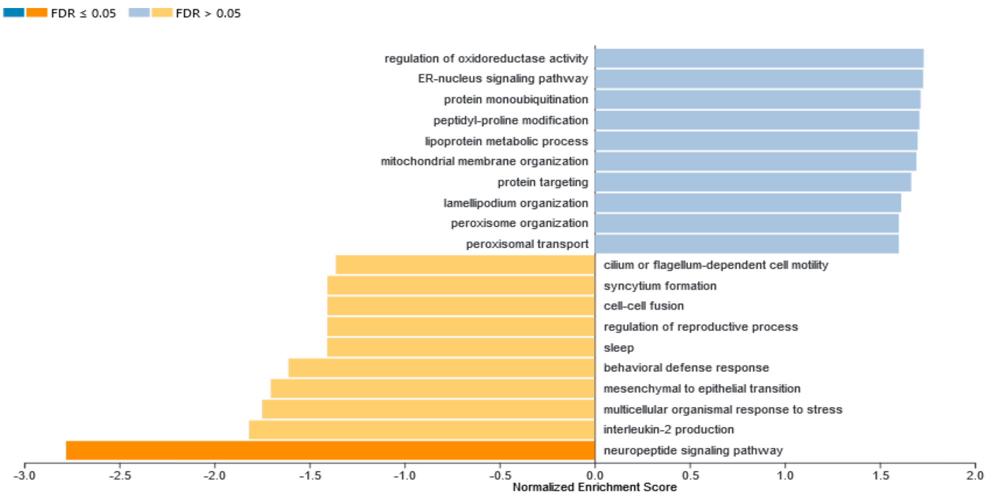

B – HNSCC tissue vs. oral lesions.

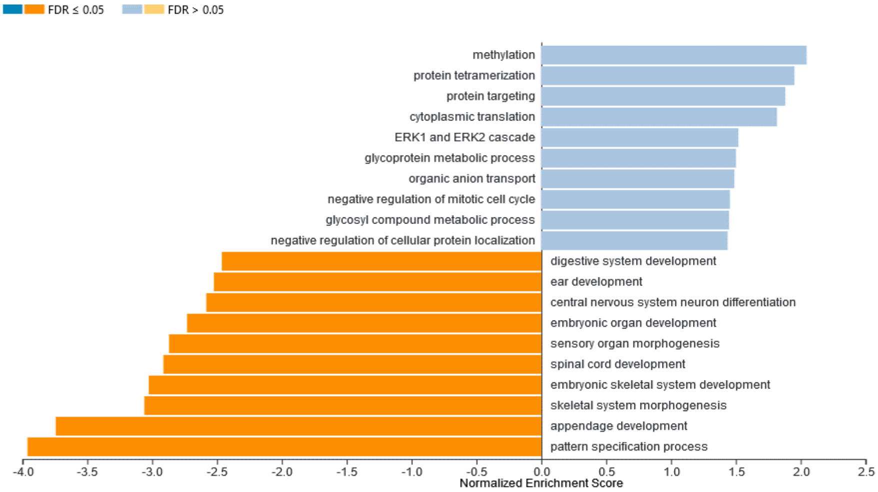

C – Oral lesions vs. healthy oral tissue.

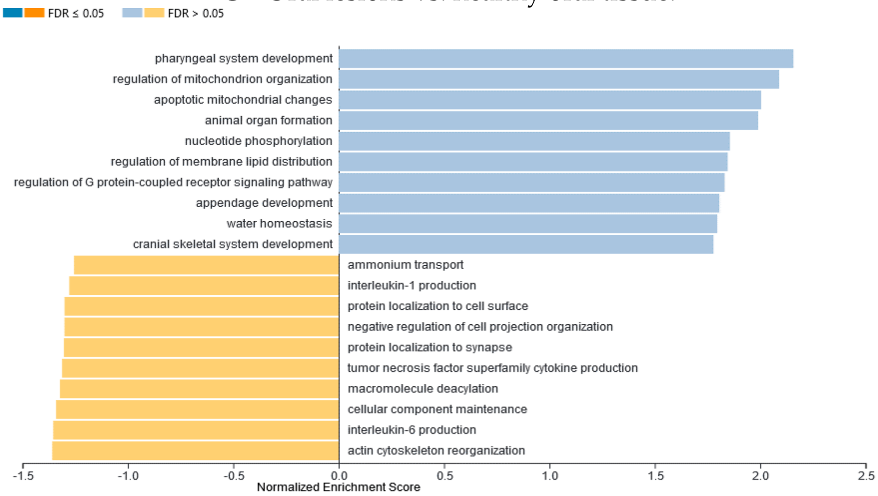

Figure S12. GSEA of GO biological processes for hypermethylated gene promoters and/or CpG sites in (**A**) HNSCC tissue compared to healthy oral tissue, (**B**) HNSCC tissue compared to potentially premalignant oral lesions, and (**C**) potentially premalignant oral lesions compared to healthy oral tissue. The top 10 categories are shown; FDR adjusted significance is indicated as the colored bar.

A – HNSCC tissue vs. healthy oral tissue.

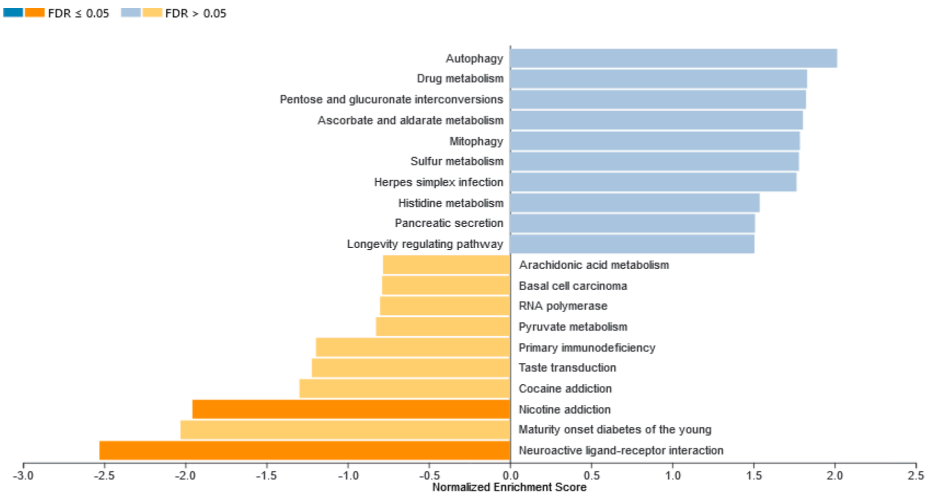

B – HNSCC tissue vs. oral lesions.

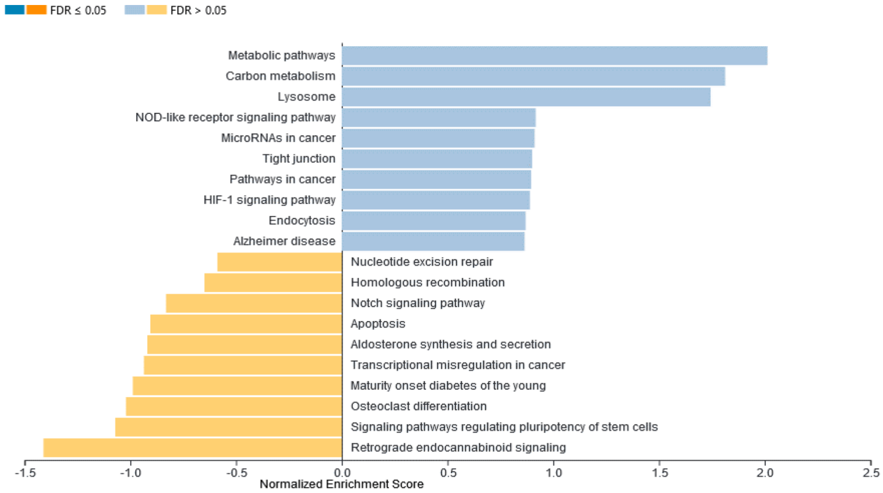

C – Oral lesions vs. healthy oral tissue.

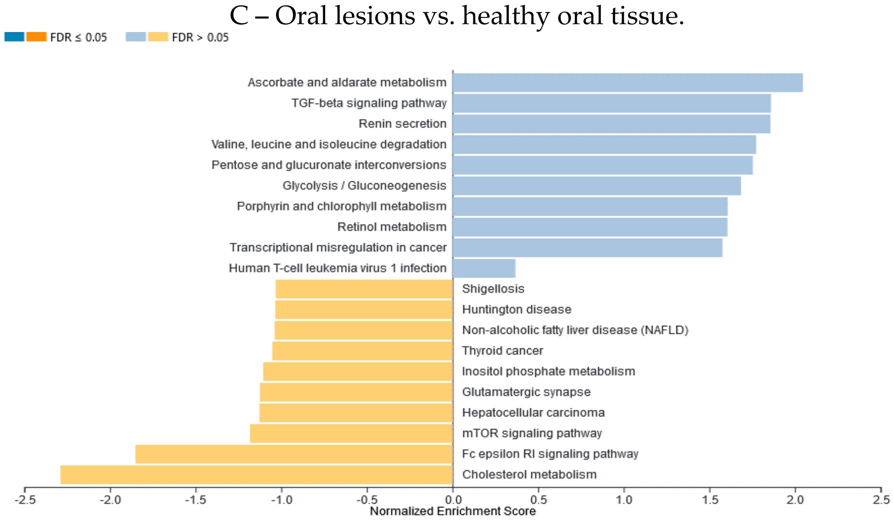

Figure A13. GSEA analysis of KEGG pathway for consistantly hypermethylated gene promoters and/or CpG sites in (**A**) HNSCC tissue compared to healthy oral tissue, (**B**) HNSCC tissue compared to potentially premalignant oral lesions, and (**C**) potentially premalignant oral lesions compared to healthy oral tissue. The top 10 categories are shown; FDR adjusted significance is indicated as the colored bar.

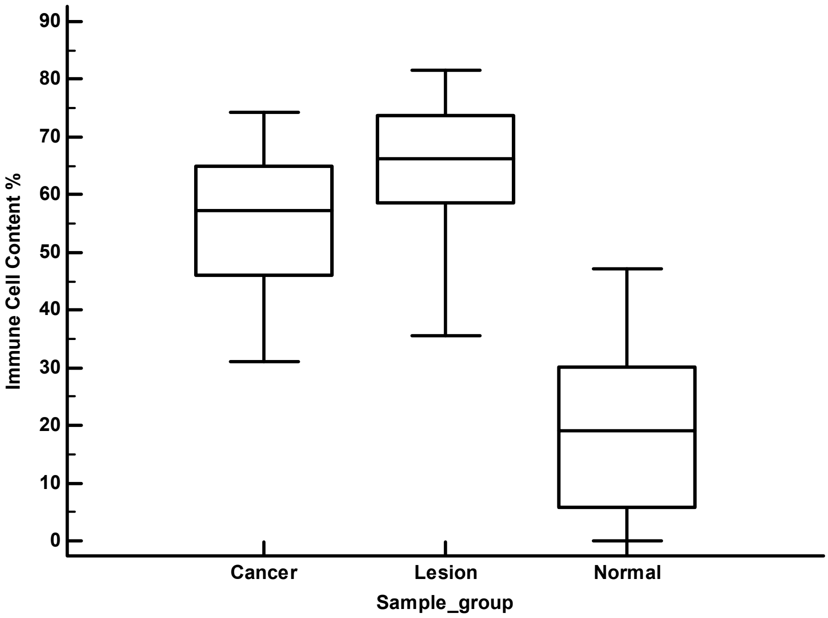

Figure S14. Average of immune cell content in different groups of samples (HNSCC, oral lesions, and healthy oral tissue), estimated by leukocytes unmethylation for purity (LUMP) method: 56% in HNSCC, 65% in potentially premalignant oral lesions, and 20% in healthy oral tissue ($p < 0.05$ Student-Newman-Keuls test).
